# Emerging Kinase Therapeutic Targets in Pancreatic Ductal Adenocarcinoma and Pancreatic Cancer Desmoplasia

**DOI:** 10.3390/ijms21228823

**Published:** 2020-11-21

**Authors:** Justin F. Creeden, Khaled Alganem, Ali S. Imami, Nicholas D. Henkel, F. Charles Brunicardi, Shi-He Liu, Rammohan Shukla, Tushar Tomar, Faris Naji, Robert E. McCullumsmith

**Affiliations:** 1 Department of Neurosciences, College of Medicine and Life Sciences, University of Toledo, Toledo, OH 43614, USA; khaled.alganem@rockets.utoledo.edu (K.A.); ali.imami@rockets.utoledo.edu (A.S.I.); nicholas.henkel@rockets.utoledo.edu (N.D.H.); rammohan.shukla@utoledo.edu (R.S.); robert.mccullumsmith@utoledo.edu (R.E.M.); 2 Department of Cancer Biology, College of Medicine and Life Sciences, University of Toledo, Toledo, OH 43614, USA; francis.brunicardi@utoledo.edu (F.C.B.); shi-he.liu@utoledo.edu (S.-H.L.); 3 Department of Surgery, College of Medicine and Life Sciences, University of Toledo, Toledo, OH 6038, USA; 4 PamGene International BV, 5200 BJ’s-Hertogenbosch, The Netherlands; ttomar@pamgene.com (T.T.); fnaji@pamgene.com (F.N.); 5 Neurosciences Institute, ProMedica, Toledo, OH 6038, USA

**Keywords:** pancreatic cancer, kinase therapy, fibrosis, desmoplasia, drug discovery, transcription factors, SRC family kinases

## Abstract

Kinase drug discovery represents an active area of therapeutic research, with previous pharmaceutical success improving patient outcomes across a wide variety of human diseases. In pancreatic ductal adenocarcinoma (PDAC), innovative pharmaceutical strategies such as kinase targeting have been unable to appreciably increase patient survival. This may be due, in part, to unchecked desmoplastic reactions to pancreatic tumors. Desmoplastic stroma enhances tumor development and progression while simultaneously restricting drug delivery to the tumor cells it protects. Emerging evidence indicates that many of the pathologic fibrotic processes directly or indirectly supporting desmoplasia may be driven by targetable protein tyrosine kinases such as Fyn-related kinase (FRK); B lymphoid kinase (BLK); hemopoietic cell kinase (HCK); ABL proto-oncogene 2 kinase (ABL2); discoidin domain receptor 1 kinase (DDR1); Lck/Yes-related novel kinase (LYN); ephrin receptor A8 kinase (EPHA8); FYN proto-oncogene kinase (FYN); lymphocyte cell-specific kinase (LCK); tec protein kinase (TEC). Herein, we review literature related to these kinases and posit signaling networks, mechanisms, and biochemical relationships by which this group may contribute to PDAC tumor growth and desmoplasia.

## 1. Introduction

Pancreatic endocrine tissue contains distinct populations of hormone-producing pancreatic islet cells. Islet α cells synthesize glucagon; islet δ cells synthesize somatostatin; islet β cells synthesize insulin. Pancreatic exocrine tissue contains acinar cells that synthesize digestive enzymes subsequently carried toward the duodenum by branched tubes lined by ductal epithelial cells. Most pancreatic cancers (approximately 95%) develop from these ductal cells. These cancers are generally called pancreatic ductal adenocarcinomas (PDAC) and present notoriously poor prognoses.

PDAC accounts for approximately 90% of all pancreatic cancers and remains resistant to treatment, in part, due to its extensive desmoplastic stroma [1]. PDAC manifests an extraordinarily dense fibrotic stroma that supports pancreatic tumor progression and metastatic spread. The extreme desmoplastic reaction to PDAC impedes nutrient and oxygen delivery to tissue and supports abnormal, oncometabolic cellular processes. This fibrosis also hinders delivery of anticancer therapeutics and facilitates drug resistance. The desmoplastic environment is beneficial to the disease and an obstruction to clinical intervention. The extracellular matrix and its constituents play important roles in the differentiation, proliferation, migration, and survival of nearby cells [2]. When the biophysical and biochemical connections between substrate and tissue become dysregulated, the stromal dynamics that normally maintain organ homeostasis break down [3]. Sufficiently distorted tissue microenvironments lose their ability to attenuate tumor development and, instead, contribute to tumor progression [4]. Tissue injury or chronic organ inflammation may serve as the impetus for this change. This desmoplastic stroma, constituting up to 90% of PDAC tumor volume [1], represents a significant challenge to pancreatic cancer therapy. These exaggerated fibrotic processes limit blood flow and drug delivery to pancreatic tumor cells while also enabling cooperative relationships between tumor cells and stromal constituents that enable malignant progression [5]. Prior reports suggest that targeting kinases involved in these unique fibrotic pathways may enhance the antitumor effects of previously established PDAC therapies [6,7,8,9,10,11].

The mortality rate of pancreatic cancer is high; more than four decades of research have improved 5-year survival rates from 4 to 7% [12]. Radiotherapy, chemotherapy, and combination therapy regimens are only minimally effective, in part due to obstacles presented by desmoplastic stroma. Efforts to develop effective pancreatic cancer therapy have employed a gamut of techniques and strategies from sophisticated pancreatic cancer surgeries to pharmaceutical modulation of cellular signaling. Therapeutic targets include components of the tumor microenvironment, pancreatic cancer stem cells, mediators of desmoplastic stromal reactions, and critical driver mutations. 

Recently, our group undertook the identification of novel target kinases for PDAC using patient-derived pancreatic cancer cell lines and wild-type pancreatic tissue specimens [13]. During our investigation, we were struck by the appearance of many kinases related to fibrosis or desmoplasia. Pancreatic cancer desmoplasia is an area of intense research interest, given its detrimental effects on patient outcomes and its role in limiting therapeutic efficacy. Below, we review the 10 protein tyrosine kinases implicated by our recent work [13] in the development, progression, or maintenance of the inflammatory stroma that is characteristic of PDAC.

## 2. Fyn-Related Kinase (FRK) 

Initially pursued as a potential tumor suppressor, more recent experimental results support the characterization of Fyn-related kinase (FRK) as an oncogene. Indeed, the emerging narrative suggests that FRK functionality is determined in an organ-specific manner [14]. In breast and brain cancer, FRK demonstrates tumor suppressive functionality [15,16,17,18,19]. In pancreatic cancer, lung cancer, and liver cancer, FRK demonstrates oncogenic functionality [20,21,22,23]. Overall, the functionality of FRK in cancer remains controversial. FRK is overrepresented in our recent kinomic investigation of pancreatic cancer, providing additional evidence for FRK as an oncogene in malignancies of pancreatic origin [13]. However, a broader interpretation of FRK literature suggests several previously reported mechanisms may complicate this narrative.

Transgenic mice with a gene of interest operating under the control of insulin promoters are frequently used to drive site-specific expression in the pancreas. The transcription factor Pancreatic and Duodenal Homeobox 1 (PDX1) is a primary activator of insulin promoter regions. While this transcription factor likely plays a role in the kinomic regulation of pancreatic cancer cells (discussed below), genetically engineered insulin promoter sequences often rely upon PDX1 to drive the expression of their downstream genes. Transgenic mice expressing FRK in this manner [24,25,26] demonstrate increased beta cell mass but also demonstrate increased islet cell death [24]. Specifically, FRK expression induces β cell proliferation in mice subjected to 60% partial pancreatectomy [24], although FRK expression enhances beta cell death in response to cytokine treatment or antineoplastic therapy [24,27]. This suggests that the duality of FRK may not be sufficiently resolved in an organ-specific manner; it may be that FRK functionality is determined by more complex biochemical relationships. Efforts to elucidate the mechanisms responsible for FRK duality involve FRK activity as a ligand, in addition to its activity as a protein kinase. This suggests that expression, regardless of subsequent kinase activity, may be integral to the role of FRK in cancer.

**Figure 1:**
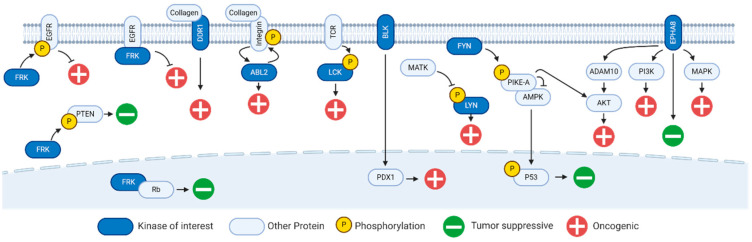
Simplified signaling pathways for kinases of interest in PDAC. Lipid bilayer represents cellular membrane; dashed line represents nuclear membrane; blue ovals represent kinases of interest; gray ovals represent other proteins; yellow circles represent phosphorylation; green circles represent downstream tumor suppression; red circles represent downstream oncogenicity or tumor promotion.

Early evidence suggests the tumor suppressor activity of FRK involves direct binding to retinoblastoma (Rb) proteins inside cellular nuclei [28] (Figure 1). Later studies indicate FRK localizes to perinuclear regions where phosphorylation inhibits cell growth independent of direct binding to Rb [15] (Figure 1). More recent evidence indicates FRK directly binds and phosphorylates the phosphatase and tensin homolog (PTEN) tumor suppressor protein, protecting PTEN from degradation and maintaining its tumor suppressive effects [16] (Figure 1). Many tumor cells for which FRK acts as a tumor suppressor are deficient in PTEN, so this relationship provides only partial explanation. Evidence supports an alternative mechanism of FRK-mediated tumor suppression in which FRK downregulates epidermal growth factor receptor (EGFR) kinase [29] (Figure 1). In normal adult tissue, EGFR regulates epithelial development. Upon ligand activation, EGFR is internalized to transduce signals from outside the plasma membrane into the intracellular space. The receptor may then be recycled to facilitate cellular proliferative processes or degraded to maintain normal cellular homeostasis [30]. FRK directly binds EGFR and may attenuate its oncogenic effects by slowing EGFR recycling [29]. FRK also phosphorylates EGFR at its Tyr^1173^ [29]. Phosphorylation of Tyr^1173^ is associated with subsequent downregulation of EGFR signaling pathways associated with tumorigenesis [31,32,33,34]. Application of these findings to our previous PDAC experimental observations suggests that increased FRK activity should correspond to decreased EGFR phosphorylation activity. Indeed, in Kinome Reverse Signature Analyzer (KRSA) [35] analyses of PANC1 cells, FRK family kinase activity demonstrates a positive log fold change (+1.6) while EGFR family kinase activity demonstrates a negative log fold change (−0.12) [13]. While there are no specific FRK tyrosine kinase inhibitors, several investigational or FDA approved drugs directly bind or otherwise inhibit FRK [36,37,38].

## 3. B Lymphoid Kinase (BLK)

B lymphoid kinase (BLK) is a nonreceptor tyrosine kinase that is preferentially expressed in B cells, and ectopically expressed in T-cell malignancies [39,40]. Preclinical studies demonstrate constitutive BLK expression increases tumor development [41] and malignant transformation [42] in lymphoma. Such evidence provides a basis for BLK as an oncogene and potential therapeutic target in blood and lymphoid tissue [43]. While the role of BLK in solid human malignancy is not as convincingly established, increased BLK activity amplifies drug resistance mechanisms in melanoma cells [44]. BLK is targeted by several anticancer drugs including ibrutinib [45], bakuchiol [46], and dasatinib [47,48]. BLK also serves as a target for additional synthetic compounds currently undergoing development [49]. Although a nascent body of evidence suggests BLK may be a useful biomarker and drug target in solid human malignancies, BLK activity is context dependent and acts as a tumor suppressor in chronic myeloid leukemia [50,51]. Recent identification of BLK as a player in the differential phosphorylation signatures of PDAC cells across three PDAC cell lines using three bioinformatic pipelines suggests BLK may play an important role in solid pancreatic malignancies. The normal physiologic role of BLK in pancreatic tissue provides additional support for this hypothesis.

In normal adult pancreatic tissue, BLK is expressed by beta cells. BLK kinase activity enhances the synthesis and secretion of insulin by upregulating the transcription factor PDX1 [52] (Figure 1). While PDX1 demonstrates protective effects during certain stages of gastric [53] and pancreatic cancer [54], it frequently switches from tumor-suppressor to oncogene after neoplastic transformation is complete [55]. We have previously proposed and investigated PDX1 as a potential target for pancreatic cancer therapy [56,57,58]; our group and others provide support for the oncogenic properties of PDX1 in pancreatic cancer and suggest additional clinical significance for PDX1 as a biomarker in other solid human malignancies such as colon, prostate, kidney, and breast cancer [59,60,61,62,63]. 

Endocrine and diabetes research provides complementary evidence of the relationship between BLK, PDX1, and pancreatic pathophysiology. PDX1, formerly known as Insulin Promoter Factor 1 (IPF1), is a transcriptional activator of insulin and plays a major role in normal glucose metabolism. Loss-of-function BLK mutations attenuate PDX1 expression to decrease insulin expression and compromise downstream signaling mechanisms [52]. In this context, recent observations suggest that increased BLK activity leads to increased PDX1 activation and enhanced insulin synthesis. The intersection of insulin signaling, diabetes, and cancer represent an active area of oncometabolic research [64,65,66,67,68,69], although the mechanistic underpinnings of these relationships remain incompletely understood. Insulin positively modulates signaling cascades that provide rapidly growing tumor cells energy and mitogenic action [70,71,72]. Many of these pro-cancer, insulin-initiated signal transduction cascades rely on the insulin receptor (INSR) tyrosine kinase family. It is noteworthy that our recent work demonstrated increased INSR family kinase activity in pancreatic cancer cells compared to wild type [13].

## 4. Hemopoietic Cell Kinase (HCK)

Like BLK, hemopoietic cell kinase (HCK) can be found in B lymphocyte lineages [73]. Although HCK is predominantly expressed in B lymphocyte progenitors, rather than in mature B lymphocyte [74], leukemias and many solid malignancies demonstrate a positive correlation between HCK activity and cancer cell proliferation and survival [75]. It may be argued that HCK is associated with hyperproliferative diseases because HCK is expressed in progenitor cells. There is emerging recognition in hematological cancer research that failure of early progenitor cells to differentiate contributes to cancer initiation and progression. HCK has previously been identified and studied as a promising therapeutic target for colon cancer [76] where HCK acts to promote tumor progression [77].

Data concerning HCK and pancreatic cancer are not abundantly represented in the literature, although one study demonstrates that gain of the HCK locus in PDAC patient biopsy predicts decreased patient survival [78]. Interestingly, HCK inhibition serves as the cornerstone of several emerging pharmacological strategies. Nonpeptide small molecule allosteric modulators using pyrimidine diamine scaffolds as HCK ligands/inhibitors were recently investigated as potential treatments for leukemia [79]. Novel immunotoxin-based strategies, in combination with drug-based inhibition or genetic reduction of HCK, have been explored in PDAC, lymphoma, and ovarian carcinoma models. Recombinant immunotoxins represent an emerging anticancer therapeutic modality reliant upon bifunctional chimeric molecules composed of a targeting domain and a cytotoxic domain. Immunotoxins are primarily used in the treatment of hematologic cancers, however, development of solid tumor immunotoxin therapies is ongoing. Previous studies indicate that the anticancer effects of an immunotoxin can be augmented by HCK inhibition [80]. Our recent results support HCK as a proto-oncogene and suggest increased focus on HCK in PDAC may be warranted. However, the role that HCK plays in pancreatic cancer may extend beyond its ostensible, direct involvement in tumor development.

Renal fibrosis is a common sequela of chronic renal allograft injury and recent work identifies HCK as an important driver of this fibrosis, with HCK overexpression activating fibrotic pathways, and HCK knockdown inhibiting fibrotic pathways [81]. Additional studies indicate HCK may play a role in other fibrotic diseases such as atherosclerosis and lung fibrosis [82,83]. Although the relationship between HCK and PDAC fibrosis or desmoplasia remains unexplored, HCK’s role in other fibrotic pathologies is suggestive of potential involvement. 

## 5. ABL Proto-Oncogene 2 Kinase (ABL2)

Leukemogenic ABL proto-oncogene 2 kinase (ABL2) fusion proteins RCSD1-ABL2 and ZC3HAV1-ABL2 have been reported in Philadelphia chromosome-like acute lymphoblastic leukemia patients [84,85]. In vivo and in vitro treatments with ABL kinase inhibitors produced cytostatic effects and in vivo, in vitro, or clinical combination therapy with dexamethasone resulted in remission [85,86]. ABL2 activity in solid tumors is not dependent upon translocation events and is instead driven by differential expression and pathophysiologic modulation of tyrosine kinase activity. Perhaps for this reason, the role of ABL2 in solid human malignancies is more paradoxical than in hematologic malignancies. In PDAC, ABL2 expression is often upregulated [87]. The implication that ABL2 is oncogenic in PDAC is supported by studies of lung cancer [88,89], hepatocellular carcinoma [90], glioma [91], and gastric cancer [92]. However, the role of ABL2 may be context dependent with ABL2 demonstrating tumor suppressive properties in some cancer models [93,94,95,96,97]. 

As discussed above, PDAC is characterized by large-scale desmoplasia. In addition to receptor tyrosine-kinase signal transduction pathways, signals from the extracellular environment may be transduced by integrin molecules that bind and activate nonreceptor tyrosine kinases. Collagens are well represented in the tumor stroma of PDAC and play significant roles in pancreatic tumor progression [98,99,100,101]. Evidence suggests that pancreatic desmoplastic protumor signaling is initiated by extracellular collagen proteins binding to intermembranous integrin molecules thereby activating cytoplasmic ABL2 nonreceptor tyrosine kinase [102,103,104,105,106,107] (Figure 1). Thus activated, ABL2′s kinase activity supports a number of downstream protumor cellular processes. Importantly, integrin-ABL2 signaling is two-way. Phosphorylated ABL2 can, in turn, phosphorylate the integrin tail and create a second binding interface for ABL2 [102]. This positive feedback likely enhances ABL2′s effects on downstream cellular behavior. In addition to tumor cell behaviors such as cell invasiveness [108], integrin-ABL2 signaling also regulates fibroblast cell behaviors such as proliferation [109], adhesion-dependent cell edge protrusion [110,111], and perhaps even activation [109]. ABL2 has been identified as a high-affinity target of dasatinib [112,113]. Dasatinib therapy enhances the inhibitory activity of paclitaxel and gemcitabine in human pancreatic cancer cells [114]. This supports our hypothesis that ABL2 is an important player in PDAC.

## 6. Discoidin Domain Receptor 1 Kinase (DDR1)

**Figure 2:**
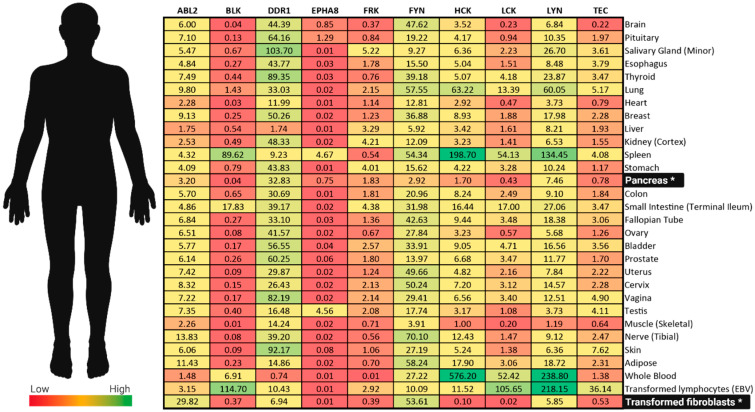
Kinase tissue distributions. The Genotype-Tissue Expression (GTEx) database was queried for all kinases of interest including Fyn-related kinase (FRK); B lymphoid kinase (BLK); hemopoietic cell kinase (HCK); ABL proto-oncogene 2 kinase (ABL2); discoidin domain receptor 1 kinase (DDR1); Lck/Yes-related novel kinase (LYN); ephrin receptor A8 kinase (EPHA8); FYN proto-oncogene kinase (FYN); lymphocyte cell-specific kinase (LCK); tec protein kinase (TEC)). Average median transcripts per million (TPM) were calculated for adipose, brain, cervix, colon, esophagus, heart, and skin subcategories. Asterisks (*) indicate key data relating to pancreatic ductal adenocarcinoma (PDAC). Samples collected from nondiseased tissue.

Another collagen receptor, discoidin domain receptor 1 (DDR1) is a receptor tyrosine kinase that belongs to a family of nonintegrin signal transducers (Figure 1). Of all the kinases discussed in this review, DDR1 kinase demonstrates the highest expression levels in normal pancreatic tissue (Figure 2). Beyond normal human physiology, increased DDR1 expression has been reported in fibrotic disease and cancer [115]. Like many of the kinases in this list of emerging kinase targets, evidence suggests DDR1 has dualistic characteristics that are context dependent. While the regulatory direction (i.e., upregulation or downregulation) varies, DDR1 is generally involved in fibrotic processes. Previous work associates DDR1 expression with poor prognosis in PDAC [115]. 

An insightful review by Moll and colleagues documents additional points that highlight the atypical landscape of current DDR1 research [116]. In brief, these include a paucity of laboratories investigating DDR1 in inflammatory cells; a string of retractions undermining aspects of the topic’s knowledge base; absence of a commercially available DDR1-specific human antibody; and—in at least one study—a striking contrast between in vitro and in vivo results. Previous reports associate upregulated DDR1 with increased metastatic potential in pancreatic cancer [117]. Activation of DDR1 contributes to tumorgenicity in PDAC [118] and selective, DDR1-specific kinase inhibitors can decrease cancer cell proliferation, invasion, and adhesion across several cancer models [118,119]. In fibrotic disease models, DDR1 inhibitors reduce inflammation and fibrosis [120,121,122,123], further positioning DDR1 as a kinase target of interest in PDAC.

## 7. Lck/Yes-Related Novel Kinase (LYN)

Desmoplasia restricts oxygen and nutrient delivery; decreased vascularization results in hypoxic collections of cells with abnormal, compensatory metabolic processes that allow surviving tumor cells to overcome otherwise uninhabitable environmental conditions [124]. Without sufficient oxygen, tumor cells switch from high-yield oxidative phosphorylation to low-yield anaerobic glycolysis. This strategy requires increased delivery of glucose to tumor cells. With blood supplies restricted, tumor cells increase glucose transporters [125]. In normal adult muscle and fat cells, insulin stimulates glucose uptake by upregulating glucose transporter 4 (GLUT4). While insulin primarily upregulates GLUT4 in normal physiologic contexts, the aberrant metabolic landscape of pancreatic cancer allows insulin to enhance GLUT1 expression [126]. In pancreatic cancer cells, upregulation of GLUT1 transporters accommodates aerobic glycolysis [127]. Lck/Yes-related novel kinase (LYN) directly phosphorylates insulin receptor 1 (IRS-1) [128], which plays a role in GLUT4 translocation [129], GLUT1 translocation [130], and appears to be required for oncometabolic changes [131]. LYN activation sensitizes insulin receptors [132,133], and increases glucose transport [128,134]. LYN is an important player in multiple tumor-related functions [135] and is upregulated in cervical cancers [136], prostate cancers [137], colon cancer [135], and Ewing’s sarcoma [138]. Further, LYN expression predicts poor prognoses for renal cancer patients [139], head and neck squamous cell carcinoma patients [140], nonsmall cell lung cancer patients [141], and breast cancer patients [142]. Phosphorylation studies (as opposed to expression studies) determined LYN kinase activity to be significantly elevated in glioblastoma [143]. Knockdown studies in pancreatic cancer cell lines demonstrate decreased pancreatic cancer cell proliferation, migration, and invasion [22] (Table 1). The mechanisms by which LYN regulates oncogenic behavior in pancreatic cancer remain incompletely understood, though several nodes in its doubtless complex signaling web have been identified and are described below.

Serotonin demonstrates vasoactive properties and promotes fibroblast activation and collagen deposition in select fibrotic contexts [144]. Serotonin is implicated in pulmonary fibrosis, [145,146,147], hepatic fibrosis [148], and renal fibrosis [149,150]. Serotonin also induces epithelial–mesenchymal transdifferentiation of renal proximal tubular epithelial cells [149] and promotes tumor growth in hepatocellular carcinoma [151]. PDAC patient tissue samples show increased serotonin concentrations relative to wild-type controls and these increased serotonin concentrations produce prosurvival and antiapoptotic effects in some pancreatic cancer cell models [152]. Protumor serotonin signaling is mediated by LYN and causes increased glucose uptake and increased anaerobic glycolysis [152]. Although serotonin does not exhibit prosurvival and antiapoptotic effects in all pancreatic cancer cell lines, these effects are observed in PANC-1 cells [152]. Consistently, our recent study identifies LYN as a lead kinase responsible for the peptide phosphorylation patterns observed in PANC-1 [13].

Alternatively, another signaling node of LYN in pancreatic cancer involves the megakaryocyte-associated tyrosine kinase (MATK) (Figure 1). This kinase is overexpressed in breast cancer patient samples and demonstrates tumor suppressive properties, inhibiting cancer cell growth and proliferation [153]. In PANC-1 cells, MATK directly phosphorylates and inhibits LYN kinase to curb pancreatic cancer cell proliferation and invasion [154]. The results from our PANC-1 and Patient-derived pancreatic ductal adenocarcinoma cell line 15 (PDCL-15) kinome arrays support and elaborate upon this observation [13]. In our experiments, the peptide sequences phosphorylated by LYN kinase are significantly overrepresented such that LYN kinase is identified as a lead candidate responsible for the overall phosphorylation patterns observed in PAN-C-1 and PDCL-15 cell lines. Furthermore, our experimental data suggests MATK kinase activity does not play a significant role in the peptide phosphorylation signatures observed in PANC-1 and PDCL-15. In other words, LYN kinase activity is appreciably upregulated when MATK kinase activity is not.

## 8. Ephrin Receptor A8 Kinase (EPHA8)

Ephrin receptor (EPH) family kinases are receptor tyrosine kinases integral to normal human development. During development, members of the EPH kinase family participate in the generation and maintenance of early vasculature and coordinate cell segregation and positioning in the gastrointestinal tract [159,160,161]. In adult tissue, members of the EPH kinase family are implicated in several solid human tumors [162,163,164,165,166]. In pancreatic cancer, evidence suggests increased EPH kinase family activity may be involved in carcinogenesis, cancer cell motility and invasion, as well as overall tumor progression and associated pain [167,168,169,170]. The EPH kinase family includes more individual kinases than any other RTK family [171,172]. Compared to other EPH kinase family members, EPHA8 is not as well studied in pancreatic cancer, and a query of PubMed using the search terms “EPHA8” and “pancreas” or “pancreatic” returns only two articles [173,174]. Of these, only one examines EPHA8 in a pancreatic cancer context [174]. The authors focus on the oncocytic subtype of intraductal papillary mucinous neoplasia of the pancreas; even when this neoplasm is associated with an invasive carcinoma, its course is less aggressive than conventional PDAC [174]. In patient samples, the authors identified two missense mutations in EPHA8 (R375H; R384H) [174]. The effects of these mutations on EPHA8 kinase activity are not immediately apparent. Increased expression of kinase-inactive EPHA8 mutants enhances cell adhesion to fibronectin proteins in the extracellular matrix [175]. These adhesions are mediated, in part, by the same integrin subunits relevant to our previous discussion of ABL2 [102,108,175] (Figure 1). Primarily a product of fibroblast cells, high concentrations of fibronectin are detected in PDAC stromal tissue, but not in normal tissue [176,177]. The biochemical signaling networks linking EPHA8 and pancreatic cancer remain unclear; follow-up studies may provide useful insights into the role that EPHA8 plays in desmoplasia. Previous investigations of EPHA8 in normal cells and tumor cells from other cancer models suggest EPHA8 likely demonstrates kinase-dependent and kinase-independent oncogenic properties. 

In fibroblasts, kinase-independent EPHA8 signaling may proceed through phosphatidylinositol 3-kinase (PI3K) mechanisms [175] (Figure 1). In neuroblastoma models, EPHA8 demonstrates kinase-independent activation of mitogen-activated protein kinase (MAPK) to promote axonal projections [178] (Figure 1). In contrast, some glioma studies suggest expression of EPHA8 suppresses migration and invasion [179]. Notably, EPHA8-mediated inhibition of cell migration does require EPHA8 kinase activity [179]. Proliferation, migration, and invasion of gastric cancer cells are associated with EPHA8 kinase-mediated signaling involving the ADAM10 protein and downstream serine/threonine kinase AKT pathways [180]. Increased EPHA8 expression also associates with increased clinicopathological features or poor prognoses in oral tongue squamous cell carcinoma [181], colorectal cancer [182], and ovarian cancer [183]. Studies of EPHA8 phosphorylation sites revealed physical associations between EPHA8 and FYN proto-oncogene kinase (FYN) and implicate FYN kinase as a major downstream target for EPHA8 signaling [184]. This observation, first made in 1999, remains largely unexplored.

## 9. FYN Proto-Oncogene Kinase (FYN)

FYN regulates downstream serine-threonine kinase activity modulating fibroblast–epithelial cell interactions and promoting organ fibrosis [185,186]. FYN signaling pathways regulate cell adhesion [187], drive epithelial-to-mesenchymal transition (EMT) [188], and play a role in migration [189], cancer cell growth and motility [190,191,192], cancer progression [193], as well as antiapoptotic activity [194]. Overall, FYN plays a significant role in the pathogenesis of many cancers [195] and varying degrees of evidence link FYN with hepatocellular carcinoma [196], oral cancer [193], mesothelioma [194], breast cancer [197], chronic myelogenous leukemia [198,199], prostate cancer [191,192], melanoma [200], brain cancer [190], and esophageal squamous cell carcinoma [201]. Evidence suggests that the role of FYN in pancreatic cancer is consistent with the role of FYN in other cancers, although its mechanism in pancreatic cancer may be somewhat unique. FYN expression is upregulated in many pancreatic cancers and its kinase activity is enhanced. In phospho-proteomic studies, activator phosphorylation sites on FYN demonstrate a two-fold increase in tumor tissue compared to wild type pancreatic patient tissue [155]. FYN knockdown or inhibition significantly reduces proliferation, migration, metastasis, and invasion in pancreatic cancer models [22,156]. Recent data suggest FYN associated pancreatic tumor pathology may depend on *KRAS* and *TP53* mutational profiles [13,202].

Foremost among potential therapeutic targets in pancreatic cancer stand cancer’s most frequently mutated oncogene family, the RAS genes [203]; and the “guardian of the genome” *TP53* tumor suppressor gene. PDAC presents with approximately 90% *KRAS* mutation frequency [204]. While there are no clinically approved drugs directly targeting KRAS, several indirect KRAS inhibitors—targeting proteins that support oncogenic KRAS functionality, rather than targeting KRAS itself—demonstrate encouraging results in other cancers yet remain unable to appreciably improve pancreatic cancer outcomes. Predictably, all patient-derived pancreatic cancer cell lines (PDCL5 and PDCL15) used in our recent study contain oncogenic *KRAS* mutations. However, PDCL5 cells express dysfunctional, mutant *TP53*, while PDCL15 cells express wild-type *TP53*. This is significant because FYN kinase is identified as a lead candidate kinase only in PDCL15 cells with functional p53 tumor suppressor proteins [13].

Mechanistic studies exploring the signaling axis of FYN in pancreatic cancer suggest significant modulation of cell cycle and apoptotic behaviors [156,157,158]. Mechanistic studies in other cancer models suggest inhibition of FYN leads to greater cell death in *KRAS* mutant cells than in *KRAS* wild-type cells [202]. Other cancer models also provide evidence that FYN phosphorylates and enhances the activity of the GTPase PIKE-A, ultimately influencing p53 behavior (Figure 1). The PIKE-A proto-oncogene directly binds and regulates two serine-threonine kinases, AMPK and AKT (Figure 1). When PIKE-A binds AMPK, it diminishes AMPK’s tumor suppression properties [205]. In the absence of PIKE-A or when AMPK is otherwise active, p53 is increasingly phosphorylated to arrest cell cycle progression [206]. In some cellular contexts, when functional p53 is not present, AMPK-dependent cell cycle arrest cannot be initiated [206]. This suggests that in *TP53* mutant pancreatic tumors, FYN-mediated AMPK suppression would impart insignificant cancer cell survival advantage. Correspondingly, FYN is not identified as a lead candidate kinase in our analyses of PDCL5 cells; these cell lines already have functionally deficient *TP53*. In contrast, our analyses did identify FYN as a candidate kinase in PDCL15 cells which express wild-type, fully functional *TP53*. Perhaps FYN demonstrates increased enzymatic activity in this cell line in order to subvert AMPK-dependent p53 tumor suppressive activity. When PIKE-A binds AKT, it enhances AKT’s antiapoptotic properties [207]. When functional p53 is present in pancreatic cancer cells, FYN-mediated AKT activation may also demonstrate antiautophagic activity. Other solid cancer cells exposed to the same nutritional insufficiencies common to pancreatic tumor cells use AKT signaling to trigger compensatory metabolic changes [208,209]. In breast cancer cell lines, AKT activates wild-type p53 proteins to protect breast cancer cells from autophagic cell death [209]. Our data implies that the oncogenic role of FYN kinase in PDAC requires mutant *KRAS* and wild-type *TP53*.

## 10. Lymphocyte Cell-Specific Kinase (LCK)

The lymphocyte cell-specific kinase (LCK) is an important regulator of T-cell functionality. Activation of the T-cell receptor (TCR) induces conformational change [210] and phosphorylation [211] of LCK (Figure 1). The enzymatic activity of LCK is critical to TCR-induced downstream activation of T cells [211]. This evidence agrees with general conceptualizations of LCK as a critical initiator of T-cell receptor signaling, as well as T-cell activation, development, and proliferation. LCK also transmits signals received by other cell receptors in T lymphocytes, natural killer lymphocytes, and B lymphocytes. Unsurprisingly, LCK has been implicated in several leukemias and immunotherapies [212]. Knowledge of the role that LCK plays in solid human tumors is developing, with reports that LCK is expressed in human breast cancer specimens [213]; LCK is overexpressed and activated in lung cancer cell lines [214]; LCK is upregulated in bile duct cancer cells and associates with early tumor recurrence [215]; LCK inhibition in human glioma cells decreases malignant progression [216]. The role that LCK plays in cancer progression may be complicated by opposing roles in tumor infiltrate and in tumor cells. LCK expression appears to be a positive prognostic marker in colorectal cancer [217] with potential to serve as a useful biomarker in early diagnosis [218]. LCK is highly expressed in subsets of primary and/or metastatic melanomas from 331 patients and associates with significantly improved survival [219]. LCK’s association with positive therapeutic outcomes in solid human cancers is likely due to immune response, rather than intrinsic cancer cell LCK abnormalities. Masitinib mesylate is a veterinary medication targeting LCK [220] that enhances Gemcitabine’s antiproliferative effects in human pancreatic cancer in in vitro and in vivo models [221]. LCK is overexpressed in pancreatic endocrine tumors, although significant associations between LCK and clinical outcomes have not been identified [222]. 

## 11. Tec Protein Kinase (TEC)

TEC family kinases are conspicuous in the development and treatment of hematogenous [223] and solid cancers including breast cancer [224], prostate cancer [225,226], liver cancer [227], glioblastoma [228], small cell lung cancer [229], colorectal cancer [230], and epithelial neoplasia [231]. The TEC family consists of TEC, BTK, ITK, TXK, and BMX nonreceptor tyrosine kinases. BTK (Bruton’s tyrosine kinase) is crucial to the oncogenic signaling pathways that drive leukemic cell survival and proliferation; BTK inhibitors demonstrate significant antitumor activity and the BTK inhibitor ibrutinib is approved as a first-line therapy in certain lymphocytic malignancies [232]. Ibrutinib also targets the TEC family member ITK (IL2 inducible T-cell kinase) and is proposed to be an effective clinical ITK inhibitor for treatment of T-cell malignancies [233]. Likewise, TEC family member TXK is implicated in T cell activation and cancer therapy [234,235,236,237,238]. While TEC family kinases are expressed in hematopoietic cells, BMX and TEC are also expressed in cells with high migratory potential [239]. BMX is expressed in cancer cells and its kinase activity is activated by extracellular matrix proteins [240]. BMX inhibition blocks integrin-mediated tumor cell migration [240]. While TEC kinase is the inaugural member of the TEC kinase family, its role in cancer pathology is incompletely understood. Overexpression of TEC kinase is reported in one study of liver cancer patients [227] but it otherwise remains largely unstudied in cancer contexts (Table 2). Recent evidence identifies TEC as a key player in the inflammatory response associated with severe pancreatitis [241]. Chronic pancreatitis is a fibrotic pathology [242] conveying increased risk of pancreatic cancer [243]. Differential phosphorylation patterns observed in pancreatic cancer imply two possible roles for TEC kinase in PDAC: increased enzymatic TEC activity may contribute to the inflammatory milieu, indirectly supporting pancreatic cancer progression; or increased enzymatic TEC activity may play a direct role, acting within the pancreatic tumor cells to activate malignant phenotypes. Further study will be required to contextualize TEC kinase activity more accurately in pancreatic cancer and desmoplasia.

## 12. Tyrosine Kinase Inhibitors

Desmoplastic stromal reactions create a biophysical barrier to drug delivery, whilst also directly promoting tumor growth and contributing to treatment resistance [244]. In the absence of sufficiently beneficial medical intervention, therapeutics that target protein tyrosine kinases represent promising drug development strategies [10]. Protein tyrosine kinases regulate key pancreatic cancer signaling pathways such as RAS-MAPK [245], PI3K-AKT [246], and JAK-STATs [247]. Inhibition of the protein tyrosine kinases that contribute to desmoplastic stromal reactions and established pancreatic cancer signaling pathways demonstrate compelling preliminary results [248,249,250]. Recent preclinical and clinical studies are rapidly contributing to our knowledge of the specific mechanisms by which individual tyrosine kinases contribute to cancer cell behavior. Collagen-induced DDR1 activation and epithelial–mesenchymal transition can be attenuated by the DDR1 inhibitor 7f which disrupts DDR1-PYK2-PEAK signaling in pancreatic cancer [251]. FRK inhibition attenuates aerobic glycolysis in some cancer cell models [23], while studies performed in other cancer cell models suggest FRK inhibition increases proliferation by downregulating the FRK-PTEN axis [252]. HCK inhibition decreases tumor growth, perhaps by impairing TGFBeta-SMAD signaling pathways [253] or STAT3-dependent tumor growth [254]. ABL2 inhibition decreases CXCL12/CXCR4-induced cancer cell invasion [91]. LYN inhibition increases cancer cell apoptosis, and reduces cancer cell proliferation, migration, and invasion, likely by inactivating the WNT/Beta-Catenin and AKT/mTOR pathways and activating the mitochondrial apoptotic pathway [135]. FYN inhibition decreases migration and invasion, possibly via STAT3 signaling [255]. Targeted inhibition of LCK results in inhibition of tumor-sphere formation in cancer stem cells [216].

Many of the inhibition studies referenced above rely upon genetic knockdown, rather than targeted inhibition of kinase activity. For pancreatic cancer, highly specific pharmaceuticals targeting single kinases (rather than families or groups of kinases) remain underdeveloped. Currently, Bosutinib [80], 7f [251], Dapagliflozin [256], Masitinib [221], and glycofullerenes [257] have been studied as potentially useful kinase inhibitors in pancreatic cancer. Alternatively, agents such as proteolysis targeting chimeras (PROTACs) degrade—rather than inhibit—protein targets and may prove better mimics of genetic knockdown for instances in which nonenzymatic activity (e.g., activity as a ligand) underlie a kinase’s role in a particular disease [258]. 

Overall, kinase inhibitors continue to show great promise in many cancers, but their contribution to improved pancreatic cancer survival has not been significant. Kinase inhibitors that attenuate the antitherapeutic characteristics of desmoplasia may play a crucial role in realizing future therapeutic efficacy. Such inhibitors may not be clinically efficacious on their own, however. Kinase inhibitors that simultaneously target desmoplastic processes and oncogenic processes would be ideal. While highly specific monotherapies have great utility as research chemicals, many kinase-controlled molecular mechanisms have robust compensatory pathways that limit the clinical impact of targeted kinase inhibition. Few absolute-selective (i.e., having a single target) small molecule inhibitors have been identified, although many dual- or multiple-target inhibitors are in use [259,260]. Polytherapy or polypharmacologic agents capable of targeting multiple components of cancer pathology are gaining traction [261,262] and may be the route of choice for future development of clinically significant anticancer drugs. 

## 13. Conclusions

These studies provide support for the validity of ongoing drug development strategies targeting protein tyrosine kinases. Our identification and contextualization (Table 1 and Table 2) of 10 kinases identified as candidate or lead candidate kinases responsible for the differential phosphorylation signatures observed between commercial and patient-derived PDAC cell lines compared to wild-type pancreatic patient samples encourages further study of the unique relationship between pancreatic tumor cells and the desmoplastic stromal environments that support tumor progression and present significant obstacles to pancreatic cancer treatment. Identification of the BLK, HCK, FRK, ABL2, DDR1, LYN, EPHA8, FYN, LCK, and TEC kinases as potentially significant mediators of pancreatic cancer progression and fibrotic development fits well into established knowledge while also advancing new avenues of investigation and discovery. Combining emerging evidence with previously published findings, we suggest a mechanism for the relationship between BLK, the PDX1 transcription factor, and pancreatic disease. This review outlines additional mechanisms by which HCK, ABL2, and DDR1 may play a role in pancreatic cancer and fibrosis. We review evidence supporting the role of LYN in oncometabolic processes and pathways by which FRK, LYN, EPHA8, and FYN may facilitate oncogenic cellular behavior. Lastly, we provide a rationale for continued investigation of the complex interplay between anticancer immune response and the activity of LCK and TEC kinases. Overall, this review identifies potential areas of investigation capable of meaningfully advancing drug development efforts targeting protein tyrosine kinases for the treatment of PDAC.

## Figures and Tables

**Table 1:** Summary table of selected kinases in pancreatic cancer.

Kinase	Study Samples	Implication	Citation
FRK	Pancreatic cancer cell lines: PANC-1 (pancreatic epithelioid carcinoma) MIA PaCa-2 (undifferentiated pancreatic carcinoma) Capan-1 (metastatic pancreatic adenocarcinoma obtained from liver) Capan-2 (pancreatic adenocarcinoma) HPAC (pancreatic adenocarcinoma).	FRK directly contributes to pancreatic cancer cell proliferation and migration in PANC-1, MIA Paca-2, Capan-1, and HPAC	[22]
HCK	Pancreatic ductal adenocarcinomas (PDACs) from 43 patients Well differentiated (*n* = 12) Moderately differentiated (*n* = 24) Poorly differentiated (*n* = 4) Lymph node metastasis Positive (*n* = 36) Negative (*n* = 6)	Gain of the HCK locus in PDAC patient biopsy predicts decreased patient survival	[78]
ABL2	22 adenocarcinoma samples 16 pancreatic cancer cell lines	In PDAC, ABL2 expression is often upregulated	[87]
DDR1	205 PDAC patient samples. T classification: T1 (*n* = 11); T2 (*n* = 31); T3 (*n* = 125); T4 (*n* = 38) *n* Classification: Absent (*n* = 136); Present (*n* =69) AJCC Stage: Stage I (*n* = 38); Stage II (*n* = 135); Stage III (*n* = 21); Stage IV (*n* = 14) Liver metastasis: Absent (*n* = 191); Present (*n* = 14)	DDR1 expression is associated with poor prognosis in PDAC	[115]
DDR1	Pancreatic cancer cell lines: PANC-1 AsPC-1 (metastatic pancreatic adenocarcinoma obtained from ascites)	Upregulated DDR1 is associated with increased metastatic potential	[117]
DDR1	Pancreatic cancer cell lines: AsPC-1 PANC-1 BxPC-3 (pancreatic adenocarcinoma) xenograft models	Activation of DDR1 contributes to tumorgenicity in PDAC. DDR1 inhibition reduces collagen-mediated tumorigenicity in PDAC	[118]
LYN	Pancreatic cancer cell lines: PANC-1 MIA PaCa-2 Capan-1 Capan-2 HPAC	LYN directly contributes to pancreatic cancer cell proliferation and migration in PANC-1, MIA Paca-2, Capan-1, Capan-2, and HPAC	[22]
FYN	12 PDAC patient samples Stage IIA (*n* = 3) Stage IIB (*n* = 9) Lymph node metastasis: Yes (*n* = 9); No (*n* = 3)	Activator phosphorylation sites on FYN demonstrate a two-fold increase in tumor tissue compared to wild type pancreatic patient tissue	[155]
FYN	Pancreatic cancer cell lines: PANC-1 MIA PaCa-2 Capan-1 Capan-2 HPAC	FYN directly contributes to pancreatic cancer cell proliferation and migration in PANC-1, MIA Paca-2, Capan-1, Capan-2, and HPAC	[22]
FYN	28 pancreatic cancer patient samples Staging: metastatic (*n* = 11); nonmetastatic (*n* = 17) TNM staging: T1 (*n* = 4); T2 (*n* = 9); T3 (*n* = 5); T4 (*n* = 10) Additional experiments were performed using BxPC3 cell lines and a nude mouse xenograft model	FYN detected in 24 (of 28) tumors Metastatic pancreatic cancer demonstrates increased FYN expression TNM staging did not correlate with FYN expression FYN inhibition reduces primary tumor weight and volume, as well as metastasis FYN inhibition decreases cell proliferation and increases apoptosis	[156]
FYN	Pancreatic cancer cell lines: BxPc3 AsPc1 PaCa2 28 pancreatic cancer patient samples TNM Staging: T1 (*n* = 4); T2 (*n* = 9); T3 (*n* = 5); T4 (*n* = 10) Staging: Nonmetastatic (*n* = 17); Metastatic (*n* = 11) Differentiation: High (*n* = 3); Middle (*n* = 16); Low (*n* = 9)	FYN activity is increased in metastatic pancreatic cancer tissue Mechanistic studies exploring the signaling axis of FYN in pancreatic cancer suggest significant coordination and regulation of apoptosis which promotes pancreatic cancer proliferation and metastasis	[157]
FYN	Pancreatic cell lines: HPDE6-C7 (immortalized human pancreatic duct epithelial cells) QGP1 (human pancreatic islet cell carcinoma) PANC-1 BxPC-3 SW1990 (metastatic pancreatic adenocarcinoma obtained from spleen) 30 pancreatic cancer patient samples TNM Staging: T1 (*n* = 4); T2 (*n* = 10); T3 (*n* = 6); T4 (*n* = 10) Staging: Nonmetastasis (*n* = 17); Metastasis (*n* = 13) Differentiation: High (*n* = 3); Middle (*n* = 15); Low (*n* = 12)	FYN mRNA expression is higher in tumor tissue compared to adjacent normal tissue FYN expression correlates with metastasis and staging	[158]

**Table 2:** Summary table of selected kinases in other contexts.

Kinase	Context	Implication	Citation
FRK	Breast cancer; brain cancer	Tumor suppressive functionality	[15,16,17,18,19]
FRK	Lung cancer; liver cancer	Oncogenic functionality	[20,21,23]
FRK	Transgenic mice	Increased expression increases beta cell mass, islet cell death, and beta cell proliferation after partial pancreatectomy	[24]
FRK	Cytokine treatment or antineoplastic therapy	Increased expression enhances beta cell death	[24,27]
FRK	Molecular	Direct binding to Rb proteins inside cellular nuclei alters Rb tumor suppressor activity	[28]
FRK	Molecular	Direct binding and phosphorylation of PTEN protects PTEN from degradation and maintains tumor suppression activity	[16]
FRK	Molecular	Direct binding of EGFR slows EGFR recycling and attenuates oncogenic effects	[29]
FRK	Molecular	Phosphorylation of EGFR downregulates tumorigenic EGFR pathways	[29,31,32,33,34]
BLK	Immune cells	Preferentially expressed in normal B cells and ectopically expressed in T cell malignancies	[39,40]
BLK	Lymphoma	Constitutive expression increases tumor development and malignant transformation	[41,42]
BLK	Melanoma cells	Amplifies drug resistance mechanisms in melanoma cells	[44]
BLK	Chronic myeloid leukemia	Tumor suppressor	[50,51]
BLK	Beta cells	Kinase activity enhances the synthesis and secretion of insulin by upregulating the transcription factor PDX1	[52]
HCK	Leukemias; solid malignancies	Positive correlation between activity and cancer cell proliferation and survival	[75]
HCK	Colon cancer	Promotes tumor progression	[77]
HCK	Immunotoxin therapy	Anticancer effects of immunotoxin are augmented by HCK inhibition	[80]
HCK	Renal fibrosis	Overexpression activates fibrotic pathways; knockdown inhibits fibrotic pathways	[81]
HCK	Atherosclerosis; lung fibrosis	Implicated in inflammatory pathways	[82,83]
ABL2	Lung cancer; hepatocellular carcinoma; glioma; gastric cancer	Oncogenic properties	[88,89,90,91,92]
ABL2	Prostate cancer; breast cancer; other cancer models	Tumor suppressive properties	[93,94,95,96,97]
ABL2	Fibroblast cells	Regulates proliferation and adhesion-dependent cell edge protrusions	[109,110,111]
DDR1	Skin; kidney; lungs	Mediates fibrotic processes in the skin; plays a protective role in the kidney and lung Expression profiles are similar in the skin and kidney, but different in the kidney and lung	[116]
DDR1	Fibrotic disease models	Inhibition reduces inflammation and fibrosis	[120,121,122,123]
LYN	Cervical cancer; prostate cancer; colon cancer; Ewing’s sarcoma	Upregulated	[135,136,137,138]
LYN	Renal cancer; head and neck squamous cell carcinoma; nonsmall cell lung cancer; breast cancer	Predicts poor prognosis	[139,140,141,142]
LYN	Glioblastoma	Kinase activity is elevated	[143]
EPHA8	Neuroblastoma	Kinase-independent activation of MAPK to promote axonal projections	[178]
EPHA8	Glioma	EPHA8-mediated inhibition of cell migration requires EPHA8 kinase activity	[179]
EPHA8	Gastric cancer	Proliferation, migration, and invasion of gastric cancer cells are associated with EPHA8 kinase-mediated signaling involving ADAM10 and downstream AKT pathways	[180]
EPHA8	Oral tongue squamous cell carcinoma; colorectal cancer; ovarian cancer	Expression associates with increased clinicopathological features or poor prognoses	[181,182,183]
FYN	Organ fibrosis	Regulates downstream serine-threonine kinase activity that modulates fibroblast–epithelial cell interactions and promotes organ fibrosis	[185,186]
FYN	Various experimental contexts	FYN signaling pathways regulate cell adhesion, drive epithelial-to-mesenchymal transition (EMT), and play a role in migration, cancer cell growth and motility; cancer progression; as well as antiapoptotic activity.	[187,188,189,190,191,192,193,194]
FYN	Hepatocellular carcinoma; oral cancer; mesothelioma; breast cancer; chronic myelogenous leukemia; prostate cancer; melanoma; brain cancer; esophageal squamous cell carcinoma	Varying degrees of evidence implicate FYN in the pathogenesis of these cancers	[190,191,192,193,194,195,196,197,198,199,200,201]
FYN	Colorectal cancer	Mechanistic studies suggest inhibition of FYN leads to greater cell death in *KRAS* mutant cells than in *KRAS* wild-type cells	[202]
LCK	T cells	Enzymatic activity is critical to TCR-induced downstream activation of T cells	[211]
LCK	Leukemia and immunotherapies	Implicated in several leukemias and immunotherapies	[212]
LCK	Breast cancer	Expressed in human breast cancer specimens	[213]
LCK	Lung cancer	Overexpressed and activated in lung cancer cell lines	[214]
LCK	Bile duct cancer	Upregulated in bile duct cancer cells and associates with early tumor recurrence	[215]
LCK	Glioma	Inhibition in human glioma cells decreases malignant progression	[216]
LCK	Colorectal cancer	Expression appears to be a positive prognostic marker; demonstrates potential as early diagnosis biomarker	[217,218]
LCK	Melanoma	Highly expressed in subsets of melanoma patients and associates with significantly improved survival	[219]
LCK	Pancreatic endocrine tumors	Overexpression	[222]
TEC	Liver cancer	Overexpression	[227]
TEC	Pancreatitis	Implicated in the inflammatory response associated with severe pancreatitis	[241]

## Abbreviations

ABL2	Abl proto-oncogene 2, nonreceptor tyrosine kinase
ADAM10	A disintegrin and metalloprotease domain 10
BLK	Blk proto-oncogene, Src family tyrosine kinase
BMX	Bmx nonreceptor tyrosine kinase
BTK	Bruton tyrosine kinase
DDR1	Discoidin domain receptor tyrosine kinase 1
EGFR	Epidermal growth factor receptor
EMT	Epithelial-to-mesenchymal transition
EPH	Ephrin receptor family kinases
EPHA8	Eph receptor A8
FRK	Fyn related Src family tyrosine kinase
FYN	FYN proto-oncogene, Src family tyrosine kinase
GLUT1	Glucose transporter 1
GLUT4	Glucose transporter 4
HCK	Hck proto-oncogene, Src family tyrosine kinase
IL2	Interleukin-2
INSR	Insulin receptor
IPF1	Insulin promoter factor 1
ITK	Il2 inducible T cell kinase
KRSA	Kinome reverse signature analyzer
LCK	Lck proto-oncogene, Src family tyrosine kinase
LYN	Lyn oroto-oncogene, Src family tyrosine kinase
MAPK	Mitogen-activated protein kinase
MATK	Megakaryocyte-associated tyrosine kinase
PDAC	Pancreatic ductal adenocarcinoma
PDCL	Patient-derived pancreatic ductal adenocarcinoma cell line
PDX1	Pancreatic and duodenal homeobox 1 transcription factor
PI3K	Phosphatidylinositol 3-kinase
PTEN	Phosphatase and tensin homolog tumor suppressor protein
Rb	Retinoblastoma
RTK	Receptor tyrosine kinase
TCR	T-cell receptor
TEC	Tec protein tyrosine kinase
TP53	Tumor protein 53
TXK	Txk tyrosine kinase

## References

[1] Orth M., Metzger P., Gerum S., Mayerle J., Schneider G., Belka C., Schnurr M., Lauber K. (2019). Pancreatic ductal adenocarcinoma: Biological hallmarks, current status, and future perspectives of combined modality treatment approaches. Radiat. Oncol..

[2] Hynes R.O. (2009). The extracellular matrix: Not just pretty fibrils. Science.

[3] Alexander J., Cukierman E. (2016). Stromal dynamic reciprocity in cancer: Intricacies of fibroblastic-ECM interactions. Curr. Opin. Cell Biol..

[4] Thomas D., Radhakrishnan P. (2019). Tumor-stromal crosstalk in pancreatic cancer and tissue fibrosis. Mol. Cancer.

[5] Melstrom L.G., Salazar M.D., Diamond D.J. (2017). The pancreatic cancer microenvironment: A true double agent. J. Surg. Oncol..

[6] Jiang H., Hegde S., Knolhoff B.L., Zhu Y., Herndon J.M., Meyer M.A., Nywening T.M., Hawkins T.M.N.W.G., Shapiro I.M., Weaver D.T. (2016). Targeting focal adhesion kinase renders pancreatic cancers responsive to checkpoint immunotherapy. Nat. Med..

[7] Wormann S.M., Song L., Ai J., Diakopoulos K.N., Kurkowski M.U., Gorgulu K., Ruess D., Campbell A., Doglioni C., Jodrell D. (2016). Loss of P53 Function Activates JAK2-STAT3 Signaling to Promote Pancreatic Tumor Growth, Stroma Modification, and Gemcitabine Resistance in Mice and Is Associated With Patient Survival. Gastroenterology.

[8] Sato T., Shibata W., Hikiba Y., Kaneta Y., Suzuki N., Ihara S., Ishii Y., Sue S., Kameta E., Sugimori M. (2017). c-Jun N-terminal kinase in pancreatic tumor stroma augments tumor development in mice. Cancer Sci..

[9] Zhang D., Li L., Jiang H., Li Q., Wang-Gillam A., Yu J., Head R., Liu J., Ruzinova M.B., Lim K.-H. (2018). Tumor-Stroma IL1beta-IRAK4 Feedforward Circuitry Drives Tumor Fibrosis, Chemoresistance, and Poor Prognosis in Pancreatic Cancer. Cancer Res..

[10] Lai E., Puzzoni M., Ziranu P., Pretta A., Impera V., Mariani S., Liscia N., Soro P., Musio F., Persano M. (2019). New therapeutic targets in pancreatic cancer. Cancer Treat. Rev..

[11] Garber K. (2010). Stromal Depletion Goes on Trial in Pancreatic Cancer. J. Natl. Cancer Inst..

[12] Yabar C.S., Winter J.M. (2016). Pancreatic Cancer: A Review. Gastroenterol. Clin. N. Am..

[13] Creeden J.F., Alganem K., Imami A.S., Brunicardi F.C., Liu S.-H., Shukla R., Tomar T., Naji F., McCullumsmith R.E. (2020). Kinome Array Profiling of Patient-Derived Pancreatic Ductal Adenocarcinoma Identifies Differentially Active Protein Tyrosine Kinases. Int. J. Mol. Sci..

[14] Goel R.K., Lukong K.E. (2016). Understanding the cellular roles of Fyn-related kinase (FRK): Implications in cancer biology. Cancer Metastasis Rev..

[15] Meyer T., Xu L., Chang J., Liu E.T., Craven R.J., Cance W. (2003). Breast cancer cell line proliferation blocked by the Src-related Rak tyrosine kinase. Int. J. Cancer.

[16] Yim E.-K., Peng G., Dai H., Hu R., Li K., Lu Y., Mills G.B., Meric-Bernstam F., Hennessy B.T., Craven R.J. (2009). Rak Functions as a Tumor Suppressor by Regulating PTEN Protein Stability and Function. Cancer Cell.

[17] Zhou X., Hua L., Zhang W., Zhu M., Shi Q., Li F., Zhang L., Song C., Yu R. (2012). FRK controls migration and invasion of human glioma cells by regulating JNK/c-Jun signaling. J. Neuro-Oncol..

[18] Hua L., Zhu M., Song X., Wang J., Fang Z., Zhang C., Shi Q., Zhan W., Wang L., Meng Q. (2014). FRK suppresses the proliferation of human glioma cells by inhibiting cyclin D1 nuclear accumulation. J. Neuro-Oncol..

[19] Shi Q., Song X., Wang J., Gu J., Zhang W., Hu J., Zhou X., Yu R. (2015). FRK inhibits migration and invasion of human glioma cells by promoting N-cadherin/beta-catenin complex formation. J. Mol. Neurosci..

[20] Chen J.S., Hung W.S., Chan H.H., Tsai S.J., Sun H.S. (2013). In silico identification of oncogenic potential of fyn-related kinase in hepatocellular carcinoma. Bioinformatics.

[21] Pilati C., Letouzé E., Nault J.-C., Imbeaud S., Boulai A., Calderaro J., Poussin K., Franconi A., Couchy G., Morcrette G. (2014). Genomic Profiling of Hepatocellular Adenomas Reveals Recurrent FRK-Activating Mutations and the Mechanisms of Malignant Transformation. Cancer Cell.

[22] Je D.W., O Y.M., Ji Y.G., Cho Y., Lee D.H. (2014). The inhibition of SRC family kinase suppresses pancreatic cancer cell proliferation, migration, and invasion. Pancreas.

[23] Zhang L., Yang Y., Chai L., Bu H., Yang Y., Huang H., Ran J., Zhu Y., Li L., Chen F. (2019). FRK plays an oncogenic role in non-small cell lung cancer by enhancing the stemness phenotype via induction of metabolic reprogramming. Int. J. Cancer.

[24] Anneren C. (2002). Dual role of the tyrosine kinase GTK and the adaptor protein SHB in beta-cell growth: Enhanced beta-cell replication after 60% pancreatectomy and increased sensitivity to streptozotocin. J. Endocrinol..

[25] Docherty H.M., Hay C.W., Ferguson L.A., Barrow J., Durward E., Docherty K. (2005). Relative contribution of PDX-1, MafA and E47/beta2 to the regulation of the human insulin promoter. Biochem. J..

[26] Glick E., Leshkowitz D., Walker M.D. (2000). Transcription Factor BETA2 Acts Cooperatively with E2A and PDX1 to Activate the Insulin Gene Promoter. J. Biol. Chem..

[27] Annerén C., Welsh M. (2001). Increased cytokine-induced cytotoxicity of pancreatic islet cells from transgenic mice expressing the Src-like tyrosine kinase GTK. Mol. Med..

[28] Craven R.J., Cance W., Liu E.T. (1995). The nuclear tyrosine kinase Rak associates with the retinoblastoma protein pRb. Cancer Res..

[29] Jin L., Craven R.J. (2014). The Rak/Frk tyrosine kinase associates with and internalizes the epidermal growth factor receptor. Oncogene.

[30] Tomas A., Futter C.E., Eden E.R. (2014). EGF receptor trafficking: Consequences for signaling and cancer. Trends Cell Biol..

[31] Keilhack H., Tenev T., Nyakatura E., Godovac-Zimmermann J., Nielsen L., Seedorf K., Böhmer F.-D. (1998). Phosphotyrosine 1173 Mediates Binding of the Protein-tyrosine Phosphatase SHP-1 to the Epidermal Growth Factor Receptor and Attenuation of Receptor Signaling. J. Biol. Chem..

[32] Emlet D.R., Moscatello D.K., Ludlow L.B., Wong A.J. (1997). Subsets of Epidermal Growth Factor Receptors during Activation and Endocytosis. J. Biol. Chem..

[33] Hsu J.-M., Chen C.-T., Chou C.-K., Kuo H.-P., Li L.-Y., Lin C.-Y., Lee H.-J., Wang Y.-N., Liu M., Liao H.-W. (2011). Crosstalk between Arg 1175 methylation and Tyr 1173 phosphorylation negatively modulates EGFR-mediated ERK activation. Nat. Cell Biol..

[34] Sigismund S., Avanzato D., Lanzetti L. (2018). Emerging functions of the EGFR in cancer. Mol. Oncol..

[35] Alganem K., Shukla R., Eby H., Abel M., Zhang X., McIntyre W.B., Lee J., Au-Yeung C., Asgariroozbehani R., Panda R. (2020). Kaleidoscope: A New Bioinformatics Pipeline Web Application for In Silico Hypothesis Exploration of Omics Signatures. bioRxiv.

[36] Guo Y., Liu Y., Hu N., Yu D., Zhou C., Shi G., Zhang B., Wei M., Liu J., Luo L. (2019). Discovery of Zanubrutinib (BGB-3111), a Novel, Potent, and Selective Covalent Inhibitor of Bruton’s Tyrosine Kinase. J. Med. Chem..

[37] Kneidinger M., Schmidt U., Rix U., Gleixner K.V., Vales A., Baumgartner C., Lupinek C., Weghofer M., Bennett K.L., Herrmann H. (2008). The effects of dasatinib on IgE receptor–dependent activation and histamine release in human basophils. Blood.

[38] Rolf M.G., Curwen J.O., Veldmanjones M.H., Eberlein C., Wang J., Harmer A., Hellawell C.J., Braddock M. (2015). In vitro pharmacological profiling of R406 identifies molecular targets underlying the clinical effects of fostamatinib. Pharmacol. Res. Perspect..

[39] Krejsgaard T., Vetter-Kauczok C.S., Woetmann A., Kneitz H., Eriksen K.W., Lovato P., Zhang Q., Wasik M.A., Geisler C., Ralfkiaer E. (2009). Ectopic expression of B-lymphoid kinase in cutaneous T-cell lymphoma. Blood.

[40] Ratner L., Rauch D., Abel H., Caruso B., Noy A., Barta S.K., Parekh S., Ramos J.C., Ambinder R., Phillips A. (2016). Dose-adjusted EPOCH chemotherapy with bortezomib and raltegravir for human T-cell leukemia virus-associated adult T-cell leukemia lymphoma. Blood Cancer J..

[41] Petersen D.L., Berthelsen J., Willerslev-Olsen A., Fredholm S., Dabelsteen S., Bonefeld C.M., Geisler C., Woetmann A. (2017). A novel BLK-induced tumor model. Tumour Biol..

[42] Malek S.N., Dordai D.I., Reim J., Dintzis H., Desiderio S. (1998). Malignant transformation of early lymphoid progenitors in mice expressing an activated Blk tyrosine kinase. Proc. Natl. Acad. Sci. USA.

[43] Petersen D.L., Krejsgaard T., Berthelsen J., Fredholm S., Willerslev-Olsen A., A Sibbesen N., Bonefeld C.M., Andersen M.H., Francavilla C., Olsen J.V. (2014). B-lymphoid tyrosine kinase (Blk) is an oncogene and a potential target for therapy with dasatinib in cutaneous T-cell lymphoma (CTCL). Leukemia.

[44] Lun X.K., Szklarczyk D., Gábor A., Dobberstein N., Zanotelli V.R.T., Saez-Rodriguez J., Von Mering C., Bodenmiller B. (2019). Analysis of the Human Kinome and Phosphatome by Mass Cytometry Reveals Overexpression-Induced Effects on Cancer-Related Signaling. Mol. Cell.

[45] Kim E., Hurtz C., Koehrer S., Wang Z., Balasubramanian S., Chang B.Y., Müschen M., Davis R.E., Burger J.A. (2017). Ibrutinib inhibits pre-BCR+ B-cell acute lymphoblastic leukemia progression by targeting BTK and BLK. Blood.

[46] Kim J.E., Kim J.H., Lee Y., Yang H., Heo Y.S., Bode A.M., Lee K.W., Dong Z. (2016). Bakuchiol suppresses proliferation of skin cancer cells by directly targeting Hck, Blk, and p38 MAP kinase. Oncotarget.

[47] Montero J.C., Seoane S., Ocaña A., Pandiella A. (2011). Inhibition of Src Family Kinases and Receptor Tyrosine Kinases by Dasatinib: Possible Combinations in Solid Tumors. Clin. Cancer Res..

[48] Chen R., Chen B.-A. (2015). The role of dasatinib in the management of chronic myeloid leukemia. Drug Des. Dev. Ther..

[49] Fallacara A.L., Passannanti R., Mori M., Iovenitti G., Musumeci F., Greco C., Crespan E., Kissova M., Maga G., Tarantelli C. (2019). Identification of a new family of pyrazolo[3,4-d]pyrimidine derivatives as multitarget Fyn-Blk-Lyn inhibitors active on B- and T-lymphoma cell lines. Eur. J. Med. Chem..

[50] Zhang H., Peng C., Hu Y., Li H., Sheng Z., Chen Y., Sullivan C., Cerny J., Hutchinson L., Higgings A. (2012). The Blk pathway functions as a tumor suppressor in chronic myeloid leukemia stem cells. Nat. Genet..

[51] Crivellaro S., Carrà G., Panuzzo C., Taulli R., Guerrasio A., Saglio G., Morotti A. (2016). The non-genomic loss of function of tumor suppressors: An essential role in the pathogenesis of chronic myeloid leukemia chronic phase. BMC Cancer.

[52] Borowiec M., Liew C.W., Thompson R., Boonyasrisawat W., Hu J., Mlynarski W.M., El Khattabi I., Kim S.H., Marselli L., Rich S.S. (2009). Mutations at the BLK locus linked to maturity onset diabetes of the young and beta-cell dysfunction. Proc. Natl. Acad. Sci. USA.

[53] Ma J., Chen M., Wang J., Xia H.H., Zhu S., Liang Y., Gu Q., Qiao L., Dai Y., Zou B. (2008). Pancreatic duodenal homeobox-1 (PDX1) functions as a tumor suppressor in gastric cancer. Carcinogenesis.

[54] Bailey P., Initiative A.P.C.G., Chang D.K., Nones K., Johns A.L., Patch A.-M., Gingras M.-C., Miller D.K., Christ A.N., Bruxner T.J.C. (2016). Genomic analyses identify molecular subtypes of pancreatic cancer. Nature.

[55] Roy N., Takeuchi K.K., Ruggeri J.M., Bailey P., Chang D., Li J., Leonhardt L., Puri S., Hoffman M.T., Gao S. (2016). PDX1 dynamically regulates pancreatic ductal adenocarcinoma initiation and maintenance. Genes Dev..

[56] Jay C.M., Ruoff C., Kumar P., Maass H., Spanhel B., Miller M., Arrington A., Montalvo N., Gresham V., Rao D.D. (2013). Assessment of intravenous pbi-shRNA PDX1 nanoparticle (OFHIRNA-PDX1) in yucatan swine. Cancer Gene Ther..

[57] Wu J.X., Liu S., Yu J., Zhou G., Rao D., Jay C.M., Kumar P., Sanchez R., Templeton N., Senzer N. (2014). Vertically integrated translational studies of PDX1 as a therapeutic target for pancreatic cancer via a novel bifunctional RNAi platform. Cancer Gene Ther..

[58] Yu J., Liu S.-H., Sanchez R., Nemunaitis J., Rozengurt E., Brunicardi F.C. (2016). PDX1 associated therapy in translational medicine. Ann. Transl. Med..

[59] Ballian N., Liu S.-H., Brunicardi F.C. (2008). Transcription factor PDX-1 in human colorectal adenocarcinoma: A potential tumor marker?. World J. Gastroenterol..

[60] Duarte-Medrano G., Lopez-Mendez I., Ramirez-Luna M.A., Valdovinos-Andraca F., Cruz-Martinez R., Medina-Vera I., Perez-Monter C., Tellez-Avila F.I. (2019). Analysis of circulating blood and tissue biopsy PDX1 and MSX2 gene expression in patients with pancreatic cancer: A case-control experimental study. Medicine.

[61] Marzioni M., Germani U., Agostinelli L., Bedogni G., Saccomanno S., Marini F., Bellentani S., Barbera C., De Minicis S., Rychlicki C. (2015). PDX-1 mRNA expression in endoscopic ultrasound-guided fine needle cytoaspirate: Perspectives in the diagnosis of pancreatic cancer. Dig. Liver Dis..

[62] Liu S.H., Patel S., Gingras M.C., Nemunaitis J., Zhou G., Chen C., Li M., Fisher W., Gibbs R., Brunicardi F.C. (2011). PDX-1: Demonstration of oncogenic properties in pancreatic cancer. Cancer.

[63] Wang X.P., Li Z.J., Magnusson J., Brunicardi F.C. (2005). Tissue MicroArray analyses of pancreatic duodenal homeobox-1 in human cancers. World J. Surg..

[64] Ray A., Alalem M., Ray B.K. (2014). Insulin signaling network in cancer. Indian J. Biochem. Biophys..

[65] Home P. (2013). Insulin therapy and cancer. Diabetes Care.

[66] Djiogue S., Kamdje A.H.N., Vecchio L., Kipanyula M.J., Farahna M., Aldebasi Y., Etet P.F. (2012). Insulin resistance and cancer: The role of insulin and IGFs. Endocr. Relat. Cancer.

[67] Bose S., Le A. (2018). Glucose Metabolism in Cancer. Adv. Exp. Med. Biol..

[68] Orgel E., Mittelman S.D. (2013). The Links Between Insulin Resistance, Diabetes, and Cancer. Curr. Diabetes Rep..

[69] Vigneri R., Goldfine I.D., Frittitta L. (2016). Insulin, insulin receptors, and cancer. J. Endocrinol. Investig..

[70] Dai L., Qi Y., Chen J., Kaczorowski D., Di W., Wang W., Xia P. (2014). Sphingosine kinase (SphK) 1 and SphK2 play equivalent roles in mediating insulin’s mitogenic action. Mol. Endocrinol..

[71] Pollak M. (2008). Insulin and insulin-like growth factor signalling in neoplasia. Nat. Rev. Cancer.

[72] Pollak M. (2012). The insulin and insulin-like growth factor receptor family in neoplasia: An update. Nat. Rev. Cancer.

[73] Liu X., Castillo J.J., Munshi M., Hunter Z., Xu L., Kofides A., Tsakmaklis N., Demos M.G., Guerrera M.L., Chan G.G. (2020). Expression of the prosurvival kinase HCK requires PAX5 and mutated MYD88 signaling in MYD88-driven B-cell lymphomas. Blood Adv..

[74] Taguchi T., Kiyokawa N., Sato N., Saito M., Fujimoto J. (2000). Characteristic expression of Hck in human B-cell precursors. Exp. Hematol..

[75] Poh A.R., O’Donoghue R.J., Ernst M. (2015). Hematopoietic cell kinase (HCK) as a therapeutic target in immune and cancer cells. Oncotarget.

[76] Poh A.R., Love C.G., Masson F., Preaudet A., Tsui C., Whitehead L., Monard S., Khakham Y., Burstroem L., Lessene G. (2017). Inhibition of Hematopoietic Cell Kinase Activity Suppresses Myeloid Cell-Mediated Colon Cancer Progression. Cancer Cell.

[77] Roseweir A.K., Powell A., Horstman S.L., Inthagard J., Park J.H., McMillan N.C., Horgan P.G., Edwards J. (2019). Src family kinases, HCK and FGR, associate with local inflammation and tumour progression in colorectal cancer. Cell. Signal..

[78] Loukopoulos P., Shibata T., Katoh H., Kokubu A., Sakamoto M., Yamazaki K., Kosuge T., Kanai Y., Hosoda F., Imoto I. (2007). Genome-wide array-based comparative genomic hybridization analysis of pancreatic adenocarcinoma: Identification of genetic indicators that predict patient outcome. Cancer Sci..

[79] Dorman H.R., Close D., Wingert B.M., Camacho C.J., Johnston P.A., Smithgall T.E. (2019). Discovery of Non-peptide Small Molecule Allosteric Modulators of the Src-family Kinase, Hck. Front. Chem..

[80] Liu X.F., Xiang L., FitzGerald D.J., Pastan I. (2014). Antitumor effects of immunotoxins are enhanced by lowering HCK or treatment with SRC kinase inhibitors. Mol. Cancer Ther..

[81] Wei C., Margulies I., Menon M.C., Zhang W., Fu J., Kidd B., Keung K.L., Woytovich C., Greene I., Xiao W. (2016). Genomic Analysis of Kidney Allograft Injury Identifies Hematopoietic Cell Kinase as a Key Driver of Renal Fibrosis. J. Am. Soc. Nephrol..

[82] Smolinska M.J., Page T.H., Urbaniak A.M., Mutch B.E., Horwood N.J. (2011). Hck Tyrosine Kinase Regulates TLR4-Induced TNF and IL-6 Production via AP-1. J. Immunol..

[83] Ernst M., Inglese M., Scholz G.M., Harder K.W., Clay F.J., Bozinovski S., Waring P., Darwiche R., Kay T., Sly P. (2002). Constitutive Activation of the Src Family Kinase Hck Results in Spontaneous Pulmonary Inflammation and an Enhanced Innate Immune Response. J. Exp. Med..

[84] Roberts K.G., Li Y., Payne-Turner D., Harvey R.C., Yang Y.-L., Pei D., McCastlain K., Ding L., Lu C., Song G. (2014). Targetable Kinase-Activating Lesions in Ph-like Acute Lymphoblastic Leukemia. N. Engl. J. Med..

[85] Roberts K.G., Yang Y.-L., Payne-Turner D., Lin W., Files J.K., Dickerson K., Gu Z., Taunton J., Janke L.J., Chen T. (2017). Oncogenic role and therapeutic targeting of ABL-class and JAK-STAT activating kinase alterations in Ph-like ALL. Blood Adv..

[86] Decool G., Domenech C., Grardel N., Plesa A., Raczkiewicz I., Ducourneau B., Ruminy P., Pages M.P., Girard S., Fenwarth L. (2019). Efficacy of Tyrosine Kinase Inhibitor Therapy in a Chemotherapy-refractory B-cell Precursor Acute Lymphoblastic Leukemia With ZC3HAV1-ABL2 Fusion. Hemasphere.

[87] Crnogorac-Jurcevic T., Efthimiou E., Nielsen T., Loader J., Terris B., Stamp G., Baron A., Scarpa A., Lemoine N.R. (2002). Expression profiling of microdissected pancreatic adenocarcinomas. Oncogene.

[88] Gu J.J., Rouse C., Xu X., Wang J., Onaitis M.W., Pendergast A.M. (2016). Inactivation of ABL kinases suppresses non-small cell lung cancer metastasis. JCI Insight.

[89] Sos M.L., Michel K., Zander T., Weiss J., Frommolt P., Peifer M., Li D., Ullrich R., Koker M., Fischer F. (2009). Predicting drug susceptibility of non–small cell lung cancers based on genetic lesions. J. Clin. Investig..

[90] Xing Q.T., Qu C.M., Wang G. (2014). Overexpression of Abl2 predicts poor prognosis in hepatocellular carcinomas and is associated with cancer cell migration and invasion. OncoTargets Ther..

[91] Chen L., Zhu M., Yu S., Hai L., Zhang L., Zhang C., Zhao P., Zhou H., Wang S., Yang X. (2020). Arg kinase mediates CXCL12/CXCR4-induced invadopodia formation and invasion of glioma cells. Exp. Cell Res..

[92] Liu Y., Shao C., Zhu L., Jiang S., Li G., Zhang W., Lin Y., Ni Y., Cao H., Shao S.H. (2018). High Expression of ABL2 Suppresses Apoptosis in Gastric Cancer. Dig. Dis. Sci..

[93] Kazi J.U., Rupar K., Marhäll A., Moharram S.A., Khanum F., Shah K., Gazi M., Nagaraj S.R.M., Sun J., Chougule R.A. (2017). ABL2 suppresses FLT3-ITD-induced cell proliferation through negative regulation of AKT signaling. Oncotarget.

[94] Qiang X.F., Zhang Z.W., Liu Q., Sun N., Pan L.L., Shen J., Li T., Yun C., Li H., Shi L.H. (2014). miR-20a promotes prostate cancer invasion and migration through targeting ABL2. J. Cell Biochem..

[95] Kain K.H., Klemke R.L. (2001). Inhibition of Cell Migration by Abl Family Tyrosine Kinases through Uncoupling of Crk-CAS Complexes. J. Biol. Chem..

[96] Peacock J.G., Miller A.L., Bradley W.D., Rodriguez O.C., Webb D.J., Koleske A.J. (2007). The Abl-related Gene Tyrosine Kinase Acts through p190RhoGAP to Inhibit Actomyosin Contractility and Regulate Focal Adhesion Dynamics upon Adhesion to Fibronectin. Mol. Biol. Cell.

[97] Gil-Henn H., Patsialou A., Wang Y., Warren M.S., Condeelis J.S., Koleske A.J. (2013). Arg/Abl2 promotes invasion and attenuates proliferation of breast cancer in vivo. Oncogene.

[98] Hamada S., Masamune A. (2018). Elucidating the link between collagen and pancreatic cancer: What’s next?. Expert Rev. Gastroenterol. Hepatol..

[99] Whatcott C.J., Diep C.H., Jiang P., Watanabe A., LoBello J., Sima C., Hostetter G., Shepard H.M., Von Hoff D.D., Han H. (2015). Desmoplasia in Primary Tumors and Metastatic Lesions of Pancreatic Cancer. Clin. Cancer Res..

[100] Zeltz C., Orgel J., Gullberg D. (2014). Molecular composition and function of integrin-based collagen glues-introducing COLINBRIs. Biochim. Biophys. Acta.

[101] Apte M.V., Park S., Phillips P.A., Santucci N., Goldstein D., Kumar R.K., Ramm G.A., Buchler M., Friess H., McCarroll J.A. (2004). Desmoplastic Reaction in Pancreatic Cancer: Role of Pancreatic Stellate Cells. Pancreas.

[102] Simpson M.A., Bradley W.D., Harburger D., Parsons M., Calderwood D.A., Koleske A.J. (2015). Direct interactions with the integrin beta1 cytoplasmic tail activate the Abl2/Arg kinase. J. Biol. Chem..

[103] Pandol S., Edderkaoui M., Gukovsky I., Lugea A., Gukovskaya A. (2009). Desmoplasia of pancreatic ductal adenocarcinoma. Clin. Gastroenterol. Hepatol..

[104] Cannon A., Thompson C., Hall B.R., Jain M., Kumar S., Batra S.K. (2018). Desmoplasia in pancreatic ductal adenocarcinoma: Insight into pathological function and therapeutic potential. Genes Cancer.

[105] Schnittert J., Bansal R., Mardhian D.F., Van Baarlen J., Östman A., Prakash J. (2019). Integrin α11 in pancreatic stellate cells regulates tumor stroma interaction in pancreatic cancer. FASEB J..

[106] Yang D., Shi J., Fu H., Wei Z., Xu J., Hu Z., Zhang Y., Yan R., Cai Q. (2016). Integrinbeta1 modulates tumour resistance to gemcitabine and serves as an independent prognostic factor in pancreatic adenocarcinomas. Tumour Biol..

[107] Yang D., Tang Y., Fu H., Xu J., Hu Z., Zhang Y., Cai Q. (2018). Integrin beta1 promotes gemcitabine resistance in pancreatic cancer through Cdc42 activation of PI3K p110beta signaling. Biochem. Biophys. Res. Commun..

[108] Beaty B.T., Sharma V.P., Bravo-Cordero J.J., Simpson M.A., Eddy R.J., Koleske A.J., Condeelis J. (2013). beta1 integrin regulates Arg to promote invadopodial maturation and matrix degradation. Mol. Biol. Cell.

[109] Torsello B., De Marco S., Bombelli S., Chisci E., Cassina V., Corti R., Bernasconi D., Giovannoni R., Bianchi C., Perego R.A. (2019). The 1ALCTL and 1BLCTL isoforms of Arg/Abl2 induce fibroblast activation and extra cellular matrix remodelling differently. Biol. Open.

[110] Lapetina S., Mader C.C., Machida K., Mayer B.J., Koleske A.J. (2009). Arg interacts with cortactin to promote adhesion-dependent cell edge protrusion. J. Cell Biol..

[111] Miller A.L., Wang Y., Mooseker M.S., Koleske A.J. (2004). The Abl-related gene (Arg) requires its F-actin—microtubule cross-linking activity to regulate lamellipodial dynamics during fibroblast adhesion. J. Cell Biol..

[112] Montenegro R., Howarth A., Ceroni A., Fedele V., Farran B., Mesquita F.P., Frejno M., Berger B.-T., Heinzlmeir S., Sailem H.Z. (2020). Identification of molecular targets for the targeted treatment of gastric cancer using dasatinib. Oncotarget.

[113] Hantschel O., Rix U., Schmidt U., Burckstummer T., Kneidinger M., Schutze G., Colinge J., Bennett K.L., Ellmeier W., Valent P. (2007). The Btk tyrosine kinase is a major target of the Bcr-Abl inhibitor dasatinib. Proc. Natl. Acad. Sci. USA.

[114] Ma L., Wei J., Su G.H., Lin J. (2019). Dasatinib can enhance paclitaxel and gemcitabine inhibitory activity in human pancreatic cancer cells. Cancer Biol. Ther..

[115] Huo Y., Yang M.-W., Liu W., Yang J., Fu X., Liu D.-J., Li J., Zhang J., Hua R., Sun Y. (2015). High expression of DDR1 is associated with the poor prognosis in Chinese patients with pancreatic ductal adenocarcinoma. J. Exp. Clin. Cancer Res..

[116] Moll S., Desmouliere A., Moeller M.J., Pache J.C., Badi L., Arcadu F., Richter H., Satz A., Uhles S., Cavalli A. (2019). DDR1 role in fibrosis and its pharmacological targeting. Biochim. Biophys. Acta Mol. Cell Res..

[117] Yang J.C., Zhang Y., He S.J., Li M.M., Cai X.L., Wang H., Xu L.M., Cao J. (2017). TM4SF1 Promotes Metastasis of Pancreatic Cancer via Regulating the Expression of DDR1. Sci. Rep..

[118] Aguilera K.Y., Huang H., Du W., Hagopian M.M., Wang Z., Hinz S., Hwang T.H., Wang H., Fleming J.B., Castrillon D.H. (2017). Inhibition of Discoidin Domain Receptor 1 Reduces Collagen-mediated Tumorigenicity in Pancreatic Ductal Adenocarcinoma. Mol. Cancer Ther..

[119] Gao M., Duan L., Luo J., Zhang L., Lu X., Zhang Y., Zhang Z., Tu Z., Xu Y., Ren X. (2013). Discovery and optimization of 3-(2-(Pyrazolo[1,5-a]pyrimidin-6-yl)ethynyl)benzamides as novel selective and orally bioavailable discoidin domain receptor 1 (DDR1) inhibitors. J. Med. Chem..

[120] Wang Z., Bian H., Bartual S.G., Du W., Luo J., Zhao H., Zhang S., Mo C., Zhou Y., Xu Y. (2016). Structure-Based Design of Tetrahydroisoquinoline-7-carboxamides as Selective Discoidin Domain Receptor 1 (DDR1) Inhibitors. J. Med. Chem..

[121] Richter H., Satz A.L., Bedoucha M., Buettelmann B., Petersen A.C., Harmeier A., Hermosilla R., Hochstrasser R., Burger D., Gsell B. (2018). DNA-Encoded Library-Derived DDR1 Inhibitor Prevents Fibrosis and Renal Function Loss in a Genetic Mouse Model of Alport Syndrome. ACS Chem. Biol..

[122] Wang Z., Zhang Y., Bartual S.G., Luo J., Xu T., Du W., Xun Q., Tu Z., Brekken R.A., Ren X. (2017). Tetrahydroisoquinoline-7-carboxamide Derivatives as New Selective Discoidin Domain Receptor 1 (DDR1) Inhibitors. ACS Med. Chem. Lett..

[123] Tao J., Zhang M., Wen Z., Wang B., Zhang L., Ou Y., Tang X., Yu X., Jiang Q. (2018). Inhibition of EP300 and DDR1 synergistically alleviates pulmonary fibrosis in vitro and in vivo. Biomed. Pharmacother..

[124] Olivares O., Vasseur S. (2015). Metabolic rewiring of pancreatic ductal adenocarcinoma: New routes to follow within the maze. Int. J. Cancer.

[125] Cameron M.E., Yakovenko A., Trevino J.G. (2018). Glucose and Lactate Transport in Pancreatic Cancer: Glycolytic Metabolism Revisited. J. Oncol..

[126] Ding X.Z., Fehsenfeld D.M., Murphy L.O., Permert J., Adrian T.E. (2000). Physiological concentrations of insulin augment pancreatic cancer cell proliferation and glucose utilization by activating MAP kinase, PI3 kinase and enhancing GLUT-1 expression. Pancreas.

[127] Kumari S., Khan S., Gupta S.C., Kashyap V.K., Yallapu M.M., Chauhan S.C., Jaggi M. (2018). MUC13 contributes to rewiring of glucose metabolism in pancreatic cancer. Oncogenesis.

[128] Muller G., Schulz A., Wied S., Frick W. (2005). Regulation of lipid raft proteins by glimepiride- and insulin-induced glycosylphosphatidylinositol-specific phospholipase C in rat adipocytes. Biochem. Pharmacol..

[129] Thirone A.C., Huang C., Klip A. (2006). Tissue-specific roles of IRS proteins in insulin signaling and glucose transport. Trends Endocrinol. Metab..

[130] Hribal M.L., Federici M., Porzio O., Lauro D., Borboni P., Accili D., Lauro R., Sesti G. (2000). The Gly-->Arg972 amino acid polymorphism in insulin receptor substrate-1 affects glucose metabolism in skeletal muscle cells. J. Clin. Endocrinol. Metab..

[131] Sakurai Y., Kubota N., Takamoto I., Obata A., Iwamoto M., Hayashi T., Aihara M., Kubota T., Nishihara H., Kadowaki T. (2017). Role of insulin receptor substrates in the progression of hepatocellular carcinoma. Sci. Rep..

[132] Ochman A.R., Lipinski C.A., Handler J.A., Reaume A.G., Saporito M.S. (2012). The Lyn kinase activator MLR-1023 is a novel insulin receptor potentiator that elicits a rapid-onset and durable improvement in glucose homeostasis in animal models of type 2 diabetes. J. Pharmacol. Exp. Ther..

[133] Lipinski C.A., Reaume A.G. (2020). High throughput in vivo phenotypic screening for drug repurposing: Discovery of MLR-1023 a novel insulin sensitizer and novel Lyn kinase activator with clinical proof of concept. Bioorg. Med. Chem..

[134] Saporito M.S., Ochman A.R., Lipinski C.A., Handler J.A., Reaume A.G. (2012). MLR-1023 is a potent and selective allosteric activator of Lyn kinase in vitro that improves glucose tolerance in vivo. J. Pharmacol. Exp. Ther..

[135] Su R., Zhang J. (2020). Oncogenic role of LYN in human gastric cancer via the Wnt/beta-catenin and AKT/mTOR pathways. Exp. Ther. Med..

[136] Liu S., Hao X., Ouyang X., Dong X., Yang Y., Yu T., Hu J., Hu L. (2016). Tyrosine kinase LYN is an oncotarget in human cervical cancer: A quantitative proteomic based study. Oncotarget.

[137] Goldenberg-Furmanov M., Stein I., Pikarsky E., Rubin H., Kasem S., Wygoda M., Weinstein I., Reuveni H., Ben-Sasson S.A. (2004). Lyn is a target gene for prostate cancer: Sequence-based inhibition induces regression of human tumor xenografts. Cancer Res..

[138] Guan H., Zhou Z., Gallick G.E., Jia S.F., Morales J., Sood A.K., Corey S.J., Kleinerman E.S. (2008). Targeting Lyn inhibits tumor growth and metastasis in Ewing’s sarcoma. Mol. Cancer Ther..

[139] Roseweir A.K., Qayyum T., Lim Z., Hammond R., MacDonald A.I., Fraser S., Oades G.M., Aitchison M., Jones R.J., Edwards J. (2016). Nuclear expression of Lyn, a Src family kinase member, is associated with poor prognosis in renal cancer patients. BMC Cancer.

[140] Mao L., Deng W.W., Yu G.T., Bu L.L., Liu J.F., Ma S.R., Wu L., Kulkarni A.B., Zhang W.F., Sun Z.J. (2017). Inhibition of SRC family kinases reduces myeloid-derived suppressor cells in head and neck cancer. Int. J. Cancer..

[141] Kim Y.J., Hong S., Sung M., Park M.J., Jung K., Noh K.-W., Oh D.-Y., Lee M.-S., Oh E., Shin Y.K. (2016). LYN expression predicts the response to dasatinib in a subpopulation of lung adenocarcinoma patients. Oncotarget.

[142] Choi Y.L., Bocanegra M., Kwon M.J., Shin Y.K., Nam S.J., Yang J.H., Kao J., Godwin A.K., Pollack J.R. (2010). LYN is a mediator of epithelial-mesenchymal transition and a target of dasatinib in breast cancer. Cancer Res..

[143] Stettner M.R., Wang W., Nabors L.B., Bharara S., Flynn D.C., Grammer J.R., Gillespie G.Y., Gladson C.L. (2005). Lyn kinase activity is the predominant cellular SRC kinase activity in glioblastoma tumor cells. Cancer Res..

[144] Dolivo D.M., Larson S.A., Dominko T. (2018). Tryptophan metabolites kynurenine and serotonin regulate fibroblast activation and fibrosis. Cell Mol. Life Sci..

[145] Tawfik M.K., Makary S. (2017). 5-HT7 receptor antagonism (SB-269970) attenuates bleomycin-induced pulmonary fibrosis in rats via downregulating oxidative burden and inflammatory cascades and ameliorating collagen deposition: Comparison to terguride. Eur. J. Pharmacol..

[146] Lofdahl A., Rydell-Tormanen K., Muller C., Martina Holst C., Thiman L., Ekstrom G., Wenglen C., Larsson-Callerfelt A.K., Westergren-Thorsson G. (2016). 5-HT2B receptor antagonists attenuate myofibroblast differentiation and subsequent fibrotic responses in vitro and in vivo. Physiol. Rep..

[147] Elaidy S.M., Essawy S.S. (2016). The antifibrotic effects of alveolar macrophages 5-HT2C receptors blockade on bleomycin-induced pulmonary fibrosis in rats. Pharmacol. Rep..

[148] Ruddell R.G., Oakley F., Hussain Z., Yeung I., Bryan-Lluka L.J., Ramm G.A., Mann D.A. (2006). A role for serotonin (5-HT) in hepatic stellate cell function and liver fibrosis. Am. J. Pathol..

[149] Erikci A., Ucar G., Yabanoglu-Ciftci S. (2016). Role of serotonin in the regulation of renal proximal tubular epithelial cells. Ren. Fail..

[150] Hamasaki Y., Doi K., Maeda-Mamiya R., Ogasawara E., Katagiri D., Tanaka T., Yamamoto T., Sugaya T., Nangaku M., Noiri E. (2013). A 5-hydroxytryptamine receptor antagonist, sarpogrelate, reduces renal tubulointerstitial fibrosis by suppressing PAI-1. Am. J. Physiol. Renal Physiol..

[151] Soll C., Jang J.H., Riener M.O., Moritz W., Wild P.J., Graf R., Clavien P.A. (2010). Serotonin promotes tumor growth in human hepatocellular cancer. Hepatology.

[152] Jiang S.H., Li J., Dong F.Y., Yang J.Y., Liu D.J., Yang X.M., Wang Y.H., Yang M.W., Fu X.L., Zhang X.X. (2017). Increased Serotonin Signaling Contributes to the Warburg Effect in Pancreatic Tumor Cells Under Metabolic Stress and Promotes Growth of Pancreatic Tumors in Mice. Gastroenterology.

[153] Zrihan-Licht S., Deng B., Yarden Y., McShan G., Keydar I., Avraham H.K. (1998). Csk Homologous Kinase, a Novel Signaling Molecule, Directly Associates with the Activated ErbB-2 Receptor in Breast Cancer Cells and Inhibits Their Proliferation. J. Biol. Chem..

[154] Fu Y., Zagozdzon R., Avraham R., Avraham H.K. (2006). CHK negatively regulates Lyn kinase and suppresses pancreatic cancer cell invasion. Int. J. Oncol..

[155] Britton D., Zen Y., Quaglia A., Selzer S., Mitra V., Lobetaner C., Jung S., Bohm G., Schmid P., Prefot P. (2014). Quantification of pancreatic cancer proteome and phosphorylome: Indicates molecular events likely contributing to cancer and activity of drug targets. PLoS ONE.

[156] Chen Z.-Y., Cai L., Bie P., Wang S.-G., Jiang Y., Dong J.-H., Li X.-W. (2010). Roles of Fyn in pancreatic cancer metastasis. J. Gastroenterol. Hepatol..

[157] Chen Z.Y., Cai L., Zhu J., Chen M., Chen J., Li Z.H., Liu X.D., Wang S.G., Bie P., Jiang P. (2011). Fyn requires HnRNPA2B1 and Sam68 to synergistically regulate apoptosis in pancreatic cancer. Carcinogenesis.

[158] Dong W., Sun S.J., Qin J.J., Liu G.M. (2020). Fyn stimulates the progression of pancreatic cancer via Fyn-GluN2b-AKT axis. Eur. Rev. Med. Pharmacol. Sci..

[159] Batlle E., Wilkinson D.G. (2012). Molecular mechanisms of cell segregation and boundary formation in development and tumorigenesis. Cold Spring Harb. Perspect. Biol..

[160] Klein R. (2012). Eph/ephrin signalling during development. Development.

[161] Pitulescu M.E., Adams R.H. (2010). Eph/ephrin molecules—A hub for signaling and endocytosis. Genes Dev..

[162] Huusko P., Ponciano-Jackson D., Wolf M., Kiefer J., O Azorsa D., Tuzmen S., Weaver D., Robbins C., Moses T., Allinen M. (2004). Nonsense-mediated decay microarray analysis identifies mutations of EPHB2 in human prostate cancer. Nat. Genet..

[163] Sulman E.P., Tang X.X., Allen C., Biegel J.A., Pleasure D.E., Brodeur G.M., Ikegaki N. (1997). ECK, a human EPH-related gene, maps to 1p36.1, a common region of alteration in human cancers. Genomics.

[164] Bardelli A., Parsons D.W., Silliman N., Ptak J., Szabo S., Saha S., Markowitz S., Willson J.K., Parmigiani G., Kinzler K.W. (2003). Mutational analysis of the tyrosine kinome in colorectal cancers. Science.

[165] Noblitt L.W., Bangari D.S., Shukla S., Knapp D.W., Mohammed S., Kinch M.S., Mittal S.K. (2004). Decreased tumorigenic potential of EphA2-overexpressing breast cancer cells following treatment with adenoviral vectors that express EphrinA1. Cancer Gene Ther..

[166] Pasquale E.B. (2005). Eph receptor signalling casts a wide net on cell behaviour. Nat. Rev. Mol. Cell Biol..

[167] Liu C., Huang H., Wang C., Kong Y., Zhang H. (2014). Involvement of ephrin receptor A4 in pancreatic cancer cell motility and invasion. Oncol. Lett..

[168] Lu Z., Zhang Y., Li Z., Yu S., Zhao G., Li M., Wang Z., Wang Q., Yang Y. (2012). Overexpression of the B-type Eph and ephrin genes correlates with progression and pain in human pancreatic cancer. Oncol. Lett..

[169] Rudno-Rudzinska J., Kielan W., Frejlich E., Kotulski K., Hap W., Kurnol K., Dzierzek P., Zawadzki M., Halon A. (2017). A review on Eph/ephrin, angiogenesis and lymphangiogenesis in gastric, colorectal and pancreatic cancers. Chin. J. Cancer Res..

[170] Giaginis C., Tsourouflis G., Zizi-Serbetzoglou A., Kouraklis G., Chatzopoulou E., Dimakopoulou K., Theocharis S.E. (2010). Clinical significance of ephrin (eph)- A1, -A2, -a4, -a5 and -a7 receptors in pancreatic ductal adenocarcinoma. Pathol. Oncol. Res..

[171] Lisabeth E.M., Falivelli G., Pasquale E.B. (2013). Eph receptor signaling and ephrins. Cold Spring Harb. Perspect. Biol..

[172] Tuzi N.L., Gullick W.J. (1994). Eph, the largest known family of putative growth factor receptors. Br. J. Cancer.

[173] Katoh M., Katoh M. (2006). Comparative integromics on Eph family. Int. J. Oncol..

[174] Basturk O., Tan M., Bhanot U., Allen P., Adsay V., Scott S.N., Shah R., Berger M.F., Askan G., Dikoglu E. (2016). The oncocytic subtype is genetically distinct from other pancreatic intraductal papillary mucinous neoplasm subtypes. Mod. Pathol..

[175] Gu C., Park S. (2001). The EphA8 receptor regulates integrin activity through p110gamma phosphatidylinositol-3 kinase in a tyrosine kinase activity-independent manner. Mol. Cell. Biol..

[176] Topalovski M., Brekken R.A. (2016). Matrix control of pancreatic cancer: New insights into fibronectin signaling. Cancer Lett..

[177] Pankov R., Yamada K.M. (2002). Fibronectin at a glance. J. Cell Sci..

[178] Gu C., Shim S., Shin J., Kim J., Park J., Han K., Park S. (2005). The EphA8 receptor induces sustained MAP kinase activation to promote neurite outgrowth in neuronal cells. Oncogene.

[179] Yan Y., Wang Q., Yan X.L., Zhang Y., Li W., Tang F., Li X., Yang P. (2015). miR-10a controls glioma migration and invasion through regulating epithelial-mesenchymal transition via EphA8. FEBS Lett..

[180] Wang Y., Zhou N., Li P., Wu H., Wang Q., Gao X., Wang X., Huang J. (2019). EphA8 acts as an oncogene and contributes to poor prognosis in gastric cancer via regulation of ADAM10. J. Cell Physiol..

[181] Liu L., Wang X., Ge W. (2018). EphA8 is a Prognostic Factor for Oral Tongue Squamous Cell Carcinoma. Med. Sci. Monit..

[182] Li X., Zhang Q., Zhao L., Jiang L., Qi A., Wei Q., Song X., Wang L., Zhang L., Zhao Y. (2020). A Combined four-mRNA Signature Associated with Lymphatic Metastasis for Prognosis of Colorectal Cancer. J. Cancer.

[183] Liu X., Xu Y., Jin Q., Wang W., Zhang S., Wang X., Zhang Y., Xu X., Huang J. (2016). EphA8 is a prognostic marker for epithelial ovarian cancer. Oncotarget.

[184] Choi S., Park S. (1999). Phosphorylation at Tyr-838 in the kinase domain of EphA8 modulates Fyn binding to the Tyr-615 site by enhancing tyrosine kinase activity. Oncogene.

[185] Lin L., Shi C., Sun Z., Le N.T., Abe J.I., Hu K. (2019). The Ser/Thr kinase p90RSK promotes kidney fibrosis by modulating fibroblast-epithelial crosstalk. J. Biol. Chem..

[186] Abe J., Berk B. (1998). Fyn-Dependent Activation of p90 Ribosomal S6 Kinase (RSK) by H2O2: New Redox Sensitive Pathway. Circulation.

[187] Calautti E., Grossi M., Mammucari C., Aoyama Y., Pirro M., Ono Y., Li J., Dotto G.P. (2002). Fyn tyrosine kinase is a downstream mediator of Rho/PRK2 function in keratinocyte cell–cell adhesion. J. Cell Biol..

[188] Gujral T.S., Chan M., Peshkin L., Sorger P.K., Kirschner M.W., MacBeath G. (2014). A noncanonical Frizzled2 pathway regulates epithelial-mesenchymal transition and metastasis. Cell.

[189] Cary L.A., Chang J.F., Guan J.L. (1996). Stimulation of cell migration by overexpression of focal adhesion kinase and its association with Src and Fyn. J. Cell Sci..

[190] Lewis-Tuffin L.J., Feathers R., Hari P., Durand N., Li Z., Rodriguez F.J., Bakken K., Carlson B., Schroeder M., Sarkaria J.N. (2015). Src family kinases differentially influence glioma growth and motility. Mol. Oncol..

[191] Jensen A.R., David S.Y., Liao C., Dai J., Keller E.T., Al-Ahmadie H., Dakin-Hache K., Usatyuk P., Sievert M.F., Paner G.P. (2011). Fyn is downstream of the HGF/MET signaling axis and affects cellular shape and tropism in PC3 cells. Clin. Cancer Res..

[192] Posadas E.M., Al-Ahmadie H., Robinson V.L., Jagadeeswaran R., Otto K., Kasza K.E., Tretiakov M., Siddiqui J., Pienta K.J., Stadler W.M. (2009). FYN is overexpressed in human prostate cancer. BJU Int..

[193] Li X., Yang Y., Hu Y., Dang D., Regezi J., Schmidt B.L., Atakilit A., Chen B., Ellis D., Ramos D.M. (2003). Alphavbeta6-Fyn signaling promotes oral cancer progression. J. Biol. Chem..

[194] Eguchi R., Kubo S., Takeda H., Ohta T., Tabata C., Ogawa H., Nakano T., Fujimori Y. (2012). Deficiency of Fyn protein is prerequisite for apoptosis induced by Src family kinase inhibitors in human mesothelioma cells. Carcinogenesis.

[195] Elias D., Ditzel H.J. (2015). Fyn is an important molecule in cancer pathogenesis and drug resistance. Pharmacol. Res..

[196] Yin L., Wang Y., Guo X., Xu C., Yu G. (2018). Comparison of gene expression in liver regeneration and hepatocellular carcinoma formation. Cancer Manag. Res..

[197] Elias D., Vever H., Laenkholm A.V., Gjerstorff M.F., Yde C.W., Lykkesfeldt A.E., Ditzel H.J. (2015). Gene expression profiling identifies FYN as an important molecule in tamoxifen resistance and a predictor of early recurrence in patients treated with endocrine therapy. Oncogene.

[198] Singh M.M., Howard A., Irwin M.E., Gao Y., Lu X., Multani A., Chandra J. (2012). Expression and activity of Fyn mediate proliferation and blastic features of chronic myelogenous leukemia. PLoS ONE.

[199] Grosso S., Puissant A., Dufies M., Colosetti P., Jacquel A., Lebrigand K., Barbry P., Deckert M., Cassuto J.P., Mari B. (2009). Gene expression profiling of imatinib and PD166326-resistant CML cell lines identifies Fyn as a gene associated with resistance to BCR-ABL inhibitors. Mol. Cancer Ther..

[200] Zhang X., Huang Z., Guo Y., Xiao T., Tang L., Zhao S., Wu L., Su J., Zeng W., Huang H. (2020). The phosphorylation of CD147 by Fyn plays a critical role for melanoma cells growth and metastasis. Oncogene.

[201] Liu D., Gao M., Wu K., Zhu D., Yang Y., Zhao S. (2019). LINC00152 facilitates tumorigenesis in esophageal squamous cell carcinoma via miR-153-3p/FYN axis. Biomed. Pharmacother..

[202] Wang Y., Lin R., Ling H., Ke Y., Zeng Y., Xiong Y., Zhou Q., Zhou F., Zhou Y. (2019). Dual inhibition of CDK4 and FYN leads to selective cell death in KRAS-mutant colorectal cancer. Signal Transduct. Target. Ther..

[203] Waters A.M., Der C.J. (2018). KRAS: The Critical Driver and Therapeutic Target for Pancreatic Cancer. Cold Spring Harb. Perspect. Med..

[204] Prior I.A., Lewis P.D., Mattos C. (2012). A comprehensive survey of Ras mutations in cancer. Cancer Res..

[205] Zhang S., Qi Q., Chan C.B., Zhou W., Chen J., Luo H.R., Appin C., Brat D.J., Ye K. (2016). Fyn-phosphorylated PIKE-A binds and inhibits AMPK signaling, blocking its tumor suppressive activity. Cell Death Differ..

[206] Jones R.G., Plas D.R., Kubek S., Buzzai M., Mu J., Xu Y., Birnbaum M.J., Thompson C.B. (2005). AMP-activated protein kinase induces a p53-dependent metabolic checkpoint. Mol. Cell.

[207] Ahn J.Y., Rong R., Kroll T.G., Van Meir E.G., Snyder S.H., Ye K. (2004). PIKE (phosphatidylinositol 3-kinase enhancer)-A GTPase stimulates Akt activity and mediates cellular invasion. J. Biol. Chem..

[208] Whiteman E.L., Cho H., Birnbaum M.J. (2002). Role of Akt/protein kinase B in metabolism. Trends Endocrinol. Metab..

[209] Sudhagar S., Sathya S., Gokulapriya G., Lakshmi B.S. (2016). AKT-p53 axis protect cancer cells from autophagic cell death during nutrition deprivation. Biochem. Biophys. Res. Commun..

[210] Stirnweiss A., Hartig R., Gieseler S., Lindquist J.A., Reichardt P., Philipsen L., Simeoni L., Poltorak M., Merten C., Zuschratter W. (2013). T cell activation results in conformational changes in the Src family kinase Lck to induce its activation. Sci. Signal..

[211] Philipsen L., Reddycherla A.V., Hartig R., Gumz J., Kastle M., Kritikos A., Poltorak M.P., Prokazov Y., Turbin E., Weber A. (2017). De novo phosphorylation and conformational opening of the tyrosine kinase Lck act in concert to initiate T cell receptor signaling. Sci. Signal..

[212] Bommhardt U.H., Schraven B., Simeoni L. (2019). Beyond TCR Signaling: Emerging Functions of Lck in Cancer and Immunotherapy. Int. J. Mol. Sci..

[213] Koster A., Landgraf S., Leipold A., Sachse R., Gebhart E., Tulusan A.H., Ronay G., Schmidt C., Dingermann T. (1991). Expression of oncogenes in human breast cancer specimens. Anticancer Res..

[214] Rupniewska E., Roy R., Mauri F.A., Liu X., Kaliszczak M., Bellezza G., Cagini L., Barbareschi M., Ferrero S., Tommasi A.M. (2018). Targeting autophagy sensitises lung cancer cells to Src family kinase inhibitors. Oncotarget.

[215] Sugihara T., Werneburg N.W., Hernandez M.C., Yang L., Kabashima A., Hirsova P., Yohanathan L., Sosa C., Truty M.J., Vasmatzis G. (2018). YAP Tyrosine Phosphorylation and Nuclear Localization in Cholangiocarcinoma Cells Are Regulated by LCK and Independent of LATS Activity. Mol. Cancer Res..

[216] Zepecki J.P., Snyder K.M., Moreno M.M., Fajardo E., Fiser A., Ness J., Sarkar A., Toms S.A., Tapinos N. (2019). Regulation of human glioma cell migration, tumor growth, and stemness gene expression using a Lck targeted inhibitor. Oncogene.

[217] Clarke C.N., Lee M.S., Wei W., Manyam G., Jiang Z.-Q., Lu Y., Morris J., Broom B., Menter D., Vilar-Sanchez E. (2017). Proteomic Features of Colorectal Cancer Identify Tumor Subtypes Independent of Oncogenic Mutations and Independently Predict Relapse-Free Survival. Ann. Surg. Oncol..

[218] Janikowska G., Janikowski T., Pyka-Pajak A., Mazurek U., Janikowski M., Gonciarz M., Lorenc Z. (2018). Potential biomarkers for the early diagnosis of colorectal adenocarcinoma—Transcriptomic analysis of four clinical stages. Cancer Biomark..

[219] Cancer Genome Atlas Network (2015). Genomic Classification of Cutaneous Melanoma. Cell.

[220] Marech I., Patruno R., Zizzo N., Gadaleta C., Introna M., Zito A.F., Gadaleta C.D., Ranieri G. (2014). Masitinib (AB1010), from canine tumor model to human clinical development: Where we are?. Crit. Rev. Oncol. Hematol..

[221] Humbert M., Casteran N., Letard S., Hanssens K., Iovanna J., Finetti P., Bertucci F., Bader T., Mansfield C.D., Moussy A. (2010). Masitinib combined with standard gemcitabine chemotherapy: In vitro and in vivo studies in human pancreatic tumour cell lines and ectopic mouse model. PLoS ONE.

[222] Capurso G., Lattimore S., Crnogorac-Jurcevic T., Panzuto F., Milione M., Bhakta V., Campanini N., Swift S.M., Bordi C., Delle Fave G. (2006). Gene expression profiles of progressive pancreatic endocrine tumours and their liver metastases reveal potential novel markers and therapeutic targets. Endocr. Relat. Cancer.

[223] Wu H., Hu C., Wang A., Weisberg E.L., Chen Y., Yun C.H., Wang W., Liu Y., Liu X., Tian B. (2016). Discovery of a BTK/MNK dual inhibitor for lymphoma and leukemia. Leukemia.

[224] Bagheri-Yarmand R., Mandal M., Taludker A.H., Wang R.A., Vadlamudi R.K., Kung H.J., Kumar R. (2001). Etk/Bmx tyrosine kinase activates Pak1 and regulates tumorigenicity of breast cancer cells. J. Biol. Chem..

[225] Dai B., Kim O., Xie Y., Guo Z., Xu K., Wang B., Kong X., Melamed J., Chen H., Bieberich C.J. (2006). Tyrosine Kinase Etk/BMX Is Up-regulated in Human Prostate Cancer and Its Overexpression Induces Prostate Intraepithelial Neoplasia in Mouse. Cancer Res..

[226] Dai B., Chen H., Guo S., Yang X., Linn D.E., Sun F., Li W., Guo Z., Xu K., Kim O. (2010). Compensatory Upregulation of Tyrosine Kinase Etk/BMX in Response to Androgen Deprivation Promotes Castration-Resistant Growth of Prostate Cancer Cells. Cancer Res..

[227] Chen C., Wang G., Zhang Z.M., Xu W., Li Q., Hu Q., Wang D., Li Z.P., Yang Z.X., Suo J.Y. (2007). The expression of Tec and the level of its phosphorylation in primary hepatic carcinomas. Zhonghua Gan Zang Bing Za Zhi.

[228] Guryanova O.A., Wu Q., Cheng L., Lathia J.D., Huang Z., Yang J., MacSwords J., Eyler C.E., McLendon R.E., Heddleston J.M. (2011). Nonreceptor Tyrosine Kinase BMX Maintains Self-Renewal and Tumorigenic Potential of Glioblastoma Stem Cells by Activating STAT3. Cancer Cell.

[229] Potter D.S., Galvin M., Brown S., Lallo A., Hodgkinson C.L., Blackhall F., Morrow C.J., Dive C. (2016). Inhibition of PI3K/BMX Cell Survival Pathway Sensitizes to BH3 Mimetics in SCLC. Mol. Cancer Ther..

[230] Potter D.S., Kelly P., Denneny O., Juvin V., Stephens L.R., Dive C., Morrow C.J. (2014). BMX acts downstream of PI3K to promote colorectal cancer cell survival and pathway inhibition sensitizes to the BH3 mimetic ABT-737. Neoplasia.

[231] Paavonen K., Ekman N., Wirzenius M., Rajantie I., Poutanen M., Alitalo K. (2004). Bmx Tyrosine Kinase Transgene Induces Skin Hyperplasia, Inflammatory Angiogenesis, and Accelerated Wound Healing. Mol. Biol. Cell.

[232] Singh S.P., Dammeijer F., Hendriks R.W. (2018). Role of Bruton’s tyrosine kinase in B cells and malignancies. Mol. Cancer.

[233] Dubovsky J.A., Beckwith K.A., Natarajan G., Woyach J.A., Jaglowski S., Zhong Y., Hessler J.D., Liu T.-M., Chang B.Y., Larkin K.M. (2013). Ibrutinib is an irreversible molecular inhibitor of ITK driving a Th1-selective pressure in T lymphocytes. Blood.

[234] Rajagopal K., Sommers C.L., Decker D.C., Mitchell E.O., Korthauer U., Sperling A.I., Kozak C.A., Love P.E., Bluestone J.A. (1999). Ribp, a Novel Rlk/Txk- and Itk-Binding Adaptor Protein That Regulates T Cell Activation. J. Exp. Med..

[235] Chamorro M., Czar M.J., Debnath J., Cheng G., Lenardo M.J., Varmus H.E., Schwartzberg P.L. (2001). Requirements for activation and RAFT localization of the T-lymphocyte kinase Rlk/Txk. BMC Immunol..

[236] Schneider H., Guerette B., Guntermann C., Rudd C.E. (2000). Resting Lymphocyte Kinase (Rlk/Txk) Targets Lymphoid Adaptor SLP-76 in the Cooperative Activation of Interleukin-2 Transcription in T-cells. J. Biol. Chem..

[237] Schaeffer E.M., Debnath J., Yap G., McVicar D., Liao X.C., Littman D.R., Sher A., Varmus H.E., Lenardo M.J., Schwartzberg P.L. (1999). Requirement for Tec Kinases Rlk and Itk in T Cell Receptor Signaling and Immunity. Science.

[238] Zhong Y., Dong S., Strattan E., Ren L., Butchar J.P., Thornton K., Mishra A., Porcu P., Bradshaw J.M., Bisconte A. (2015). Targeting Interleukin-2-inducible T-cell Kinase (ITK) and Resting Lymphocyte Kinase (RLK) Using a Novel Covalent Inhibitor PRN694. J. Biol. Chem..

[239] Felices M., Falk M., Kosaka Y., Berg L.J. (2007). Tec Kinases in T Cell and Mast Cell Signaling. Advances in Immunology.

[240] Chen R., Kim O., Li M., Xiong X., Guan J.-L., Kung H.-J., Chen H., Shimizu Y., Qiu Y. (2001). Regulation of the PH-domain-containing tyrosine kinase Etk by focal adhesion kinase through the FERM domain. Nat. Cell Biol..

[241] Meng Y., Sha S., Yang J., Ren H. (2019). Effects of Tec Tyrosine Kinase Inhibition on the Inflammatory Response of Severe Acute Pancreatitis-Associated Acute Lung Injury in Mice. Dig. Dis. Sci..

[242] Whitcomb D.C., Frulloni L., Garg P., Greer J.B., Schneider A., Yadav D., Shimosegawa T. (2016). Chronic pancreatitis: An international draft consensus proposal for a new mechanistic definition. Pancreatology.

[243] Raimondi S., Lowenfels A.B., Morselli-Labate A.M., Maisonneuve P., Pezzilli R. (2010). Pancreatic cancer in chronic pancreatitis; aetiology, incidence, and early detection. Best Pr. Res. Clin. Gastroenterol..

[244] Aslan M., Shahbazi R., Ulubayram K., Ozpolat B. (2018). Targeted Therapies for Pancreatic Cancer and Hurdles Ahead. Anticancer Res..

[245] Valencia-Sama I., Ladumor Y., Kee L., Adderley T., Christopher G., Robinson C.M., Kano Y., Ohh M., Irwin M.S. (2020). NRAS Status Determines Sensitivity to SHP2 Inhibitor Combination Therapies Targeting the RAS-MAPK Pathway in Neuroblastoma. Cancer Res..

[246] Beyens M., Vandamme T., Peeters M., Van Camp G., De Beeck K.O. (2019). Resistance to targeted treatment of gastroenteropancreatic neuroendocrine tumors. Endocr. Relat. Cancer.

[247] Hu H., Zhang Q., Chen W., Wu T., Liu S., Li X., Luo B., Zhang T., Yan G., Lu H. (2020). MicroRNA-301a promotes pancreatic cancer invasion and metastasis through the JAK/STAT3 signaling pathway by targeting SOCS5. Carcinogenesis.

[248] Hosein A.N., Brekken R.A., Maitra A. (2020). Pancreatic cancer stroma: An update on therapeutic targeting strategies. Nat. Rev. Gastroenterol. Hepatol..

[249] Huang H., Wright S., Zhang J., Brekken R.A. (2019). Getting a grip on adhesion: Cadherin switching and collagen signaling. Biochim. Biophys. Acta Mol. Cell Res..

[250] Du W., Huang H., Sorrelle N., Brekken R.A. (2018). Sitravatinib potentiates immune checkpoint blockade in refractory cancer models. JCI Insight.

[251] Zhu D., Huang H., Pinkas D.M., Luo J., Ganguly D., Fox A.E., Arner E., Xiang Q., Tu Z.C., Bullock A.N. (2019). 2-Amino-2,3-dihydro-1H-indene-5-carboxamide-Based Discoidin Domain Receptor 1 (DDR1) Inhibitors: Design, Synthesis, and in Vivo Antipancreatic Cancer Efficacy. J. Med. Chem..

[252] Jing Z.F., Bi J.B., Li Z.L., Liu X.K., Li J., Zhu Y.Y., Zhang X.T., Zhang Z., Li Z.H., Kong C.Z. (2019). miR-19 promotes the proliferation of clear cell renal cell carcinoma by targeting the FRK-PTEN axis. OncoTargets Ther..

[253] Wang Z., Ying C., Zhang A., Xu H., Jiang Y., Lou M. (2020). HCK promotes glioblastoma progression by TGFbeta signaling. Biosci. Rep..

[254] Poh A.R., Dwyer A.R., Eissmann M.F., Chand A.L., Baloyan D., Boon L., Murrey M.W., Whitehead L., O’Brien M., Lowell C.A. (2020). Inhibition of the SRC Kinase HCK Impairs STAT3-Dependent Gastric Tumor Growth in Mice. Cancer Immunol. Res..

[255] Yu J., Zhou Z., Wei Z., Wu J., Ouyang J., Huang W., He Y., Zhang C. (2020). FYN promotes gastric cancer metastasis by activating STAT3-mediated epithelial-mesenchymal transition. Transl. Oncol..

[256] Okada J., Yamada E., Saito T., Yokoo H., Osaki A., Shimoda Y., Ozawa A., Nakajima Y., Pessin J.E., Okada S. (2020). Dapagliflozin Inhibits Cell Adhesion to Collagen I and IV and Increases Ectodomain Proteolytic Cleavage of DDR1 by Increasing ADAM10 Activity. Molecules.

[257] Serda M., Malarz K., Mrozek-Wilczkiewicz A., Wojtyniak M., Musiol R., Curley S.A. (2020). Glycofullerenes as non-receptor tyrosine kinase inhibitors—towards better nanotherapeutics for pancreatic cancer treatment. Sci. Rep..

[258] Sun X., Gao H., Yang Y., He M., Wu Y., Song Y., Tong Y., Rao Y. (2019). PROTACs: Great opportunities for academia and industry. Signal Transduct. Target. Ther..

[259] Wu P., Nielsen T.E., Clausen M.H. (2015). FDA-approved small-molecule kinase inhibitors. Trends Pharmacol. Sci..

[260] Davis M.I., Hunt J.P., Herrgard S., Ciceri P., Wodicka L.M., Pallares G., Hocker M., Treiber D.K., Zarrinkar P.P. (2011). Comprehensive analysis of kinase inhibitor selectivity. Nat. Biotechnol..

[261] Chatterjee N., Bivona T.G. (2019). Polytherapy and Targeted Cancer Drug Resistance. Trends Cancer.

[262] Konieczkowski D.J., Johannessen C.M., Garraway L.A. (2018). A Convergence-Based Framework for Cancer Drug Resistance. Cancer Cell.

